# Is TREM2 a Stretch: Implications of TREM2 Along Spinal Cord Circuits in Health, Aging, Injury, and Disease

**DOI:** 10.3390/cells14191520

**Published:** 2025-09-29

**Authors:** Tana S. Pottorf, Elizabeth L. Lane, Francisco J. Alvarez

**Affiliations:** 1 Department of Cell Biology, Emory University, Atlanta, GA 30322, USA; pottorf.5@osu.edu (T.S.P.); elizabeth.louise.lane@emory.edu (E.L.L.); 2 Department of Neuroscience, Ohio State University, Columbus, OH 43085, USA

**Keywords:** neuroinflammation, Triggering Receptor Expressed on Myeloid Cells 2 (TREM2), spinal reflexes, microglia, macrophage, Motor Neuron Disease (MND), peripheral nerve injury (PNI), spinal cord injury (SCI), peripheral neuropathy

## Abstract

Triggering Receptor Expressed on Myeloid Cells 2 (TREM2) is a receptor found in microglia within the central nervous system (CNS) as well as in several other cell types throughout the body. TREM2 has been highlighted as a “double-edged sword” due to its contribution to anti- or pro-inflammatory signaling responses in a spatial, temporal, and disease-specific fashion. Many of the functions of TREM2 in relation to neurological disease have been elucidated in a variety of CNS pathologies, including neurodegenerative, traumatic, and vascular injuries, as well as autoimmune diseases. Less is known about the function of TREM2 in motoneurons and sensory neurons, whose cell bodies and axons span both the CNS and peripheral nervous system (PNS) and are exposed to a variety of TREM2-expressing cells and mechanisms. In this review, we provide a brief overview of TREM2 and then highlight the literature detailing the involvement of TREM2 along the spinal cord, peripheral nerves and muscles, and sensory, motor, and autonomic functions in health, aging, disease, and injury. We further discuss the current feasibility of TREM2 as a potential therapeutic target to ameliorate damage in the sensorimotor circuits of the spinal cord.

## 1. Introduction

The spinal cord contains the central projections of sensory afferents carrying information from the body, as well as the cell bodies and dendrites of somatic and visceral autonomic motoneurons that regulate motor function in somatic and visceral muscles, respectively, by sending axons into the periphery. These neural elements interact with a variety of spinal interneurons that form local circuits to control the transfer of sensory information through the spinal cord to other CNS centers, interpret brain descending commands to drive movement of the skeletomotor system or control visceral organs (e.g., eyes, heart, gastrointestinal motility, bladder function, etc.). Within the spinal cord, some circuits organize reflexes—rapid, involuntary motor responses to sensory inputs—that can occur independently of the brain, although they are also heavily modulated by descending brain projections. Proper function of spinal reflex arcs triggered by nociceptive, mechanoreceptive, proprioceptive, and visceral receptors is key for homeostatic bodily function, movement coordination, and protection from damaging stimuli.

Motor Neuron Disease (MND), Amyotrophic Lateral Sclerosis (ALS), Spinal Muscular Atrophy (SMA), peripheral neuropathies, and injuries to the spinal cord or peripheral nerves can have significant detrimental effects on motor, sensory, and proprioceptive functions, all of which involve some degree of neuroinflammation. For example, in ALS, selective but progressive motoneuron degeneration occurs, accompanied by activated glia and infiltration of inflammatory T cells into the CNS, as well as activated monocytes and macrophages in the periphery [1,2,3]. Spinal cord injury causes direct damage to neural circuitry at the lesion zone but can also induce plasticity at lesion-remote zones [4]. Immediately following spinal cord injury, a patient may experience “spinal shock,” during which stretch reflex circuits are suppressed or absent. After spinal shock, surviving circuitry may result in hyperactivity, ultimately leading to spasticity [5]. Activation of glia and infiltration of peripheral immune cells occur at the lesion zone, but systemic immune dysfunction has also been observed, leading to secondary injuries [6,7,8]. Peripheral nerve injuries cause direct damage to motor and sensory neurons, leading to axon degeneration, cell death, spinal cord plasticity, hyperalgesia, permanent loss of stretch reflexes, and neuroinflammation at the site of the injury in the periphery as well as in the spinal cord [9]. The consequences of neuroinflammation vary from beneficial to detrimental, depending on the disease/injury, the intensity of inflammation, and how long inflammation persists. Generally, the neuroimmune field has pushed for cell-type-specific therapies in an attempt to reduce the amount of pro-inflammatory cells or increase anti-inflammatory cells in a temporally and spatially specific manner, with the ultimate goal of slowing disease progression and/or improving functional recovery following injury or insult.

**Figure 1:**
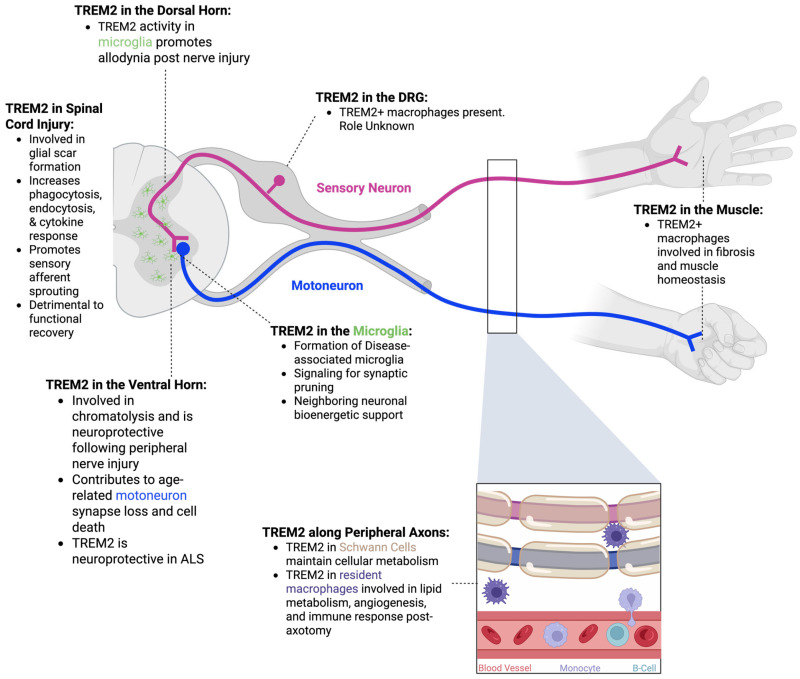
Summary of TREM2 sites of actions within sensorimotor circuits of the spinal cord, peripheral nerves, and muscles.

One potential target for cell-type-specific modification is Triggering Receptor Expressed on Myeloid Cells 2 (TREM2). TREM2 is found in both CNS and peripheral immune cells and is regarded as a “double-edged sword,” since knock-out rodent models have yielded beneficial or detrimental results depending on the disease model [10]. In this review, we provide a brief introduction to TREM2, followed by an examination of its potential implications in health, disease, and injury, utilizing the spinal cord and peripheral nervous system (PNS) anatomy as a roadmap for discussing TREM2 involvement in sensory and motor neuropathologies of the spinal cord specifically (summarized in Figure 1).

## 2. Triggering Receptor Expressed on Myeloid Cells 2 (TREM2)

### 2.1. What and Where Is TREM2?

**Figure 2:**
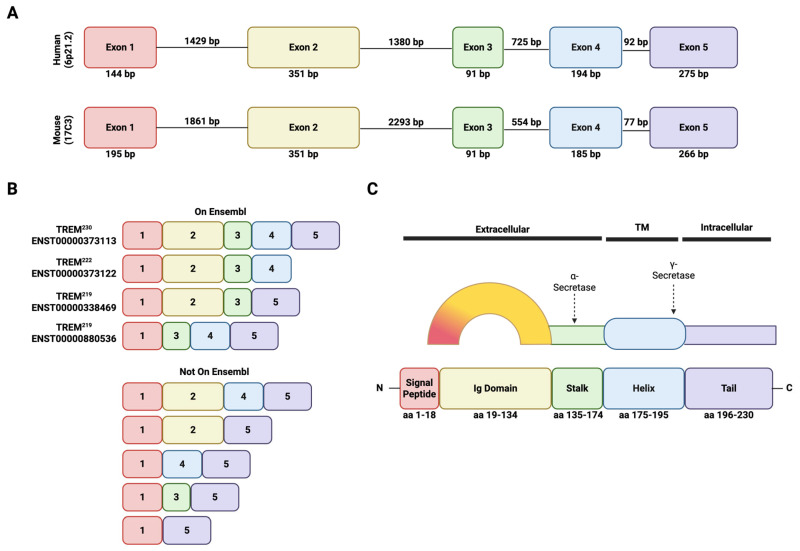
*TREM2* gene structure, splice variants, and translation. (**A**) Gene structure of *TREM2* in humans and mice. (**B**) Identified splice variants available on Ensembl or in publications [21]. (**C**) Human TREM2 protein domains. Abbreviations: bp (base pairs), aa (amino acids), and TM (transmembrane).

**Figure 3:**
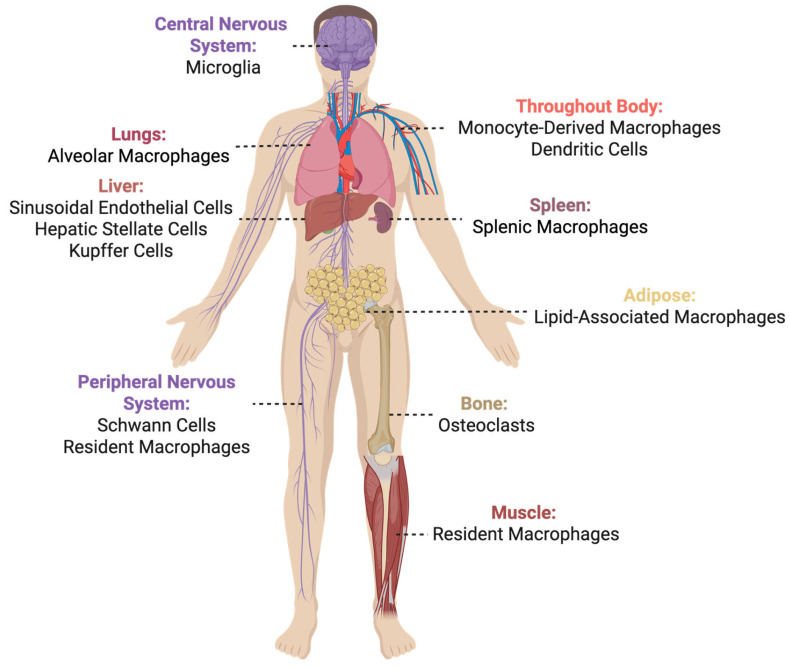
Cells that express TREM2 throughout the body.

TREM2 belongs to a family of transmembrane glycoprotein cell-surface receptors expressed by immune cells, including microglia. The TREM family has been extensively reviewed previously [11,12]. Here, we provide a brief introduction to TREM2 specifically. The functional TREM2 protein comprises an extracellular N’-terminal receptor (IgV) domain, tethered to the transmembrane domain via a stalk, and a C’-terminal cytoplasmic domain [11,13]. TREM2 lacks a signaling motif and requires an adapter protein, such as DNAX-activating protein of 12 kDa or 10 kDa (DAP_12_ or DAP_10_), encoded by *Tyrobp* or *Hcst*, respectively, to initiate a signaling cascade [14]. The *TREM2* gene is located on chromosome 6 (6p21.1) in humans and chromosome 17C in mice and is evolutionarily conserved [11,12,15,16,17,18]. In humans, *TREM2* has five exons with up to nine alternative splice transcripts [19,20,21]. The full-length human TREM2 variant, which functions as a transmembrane receptor, results in 230 amino acids (227aa in mouse), whereby amino acids (aa) 1–18 is the signaling peptide, 19- 134aa is the extracellular receptor domain, 175- 195aa is the transmembrane domain, and 196- 230aa is the intracellular domain [13,22,23,24] (Figure 2). TREM2 is expressed by microglia in the CNS; Schwann cells and resident macrophages in the PNS [25]; Kupffer cells, hepatic stellate cells, and sinusoidal endothelial cells in the liver; splenic macrophages in the spleen; alveolar macrophages in the lungs; osteoclasts in bone; dendritic cells and monocyte-derived macrophages (e.g., lipid-associated macrophages in adipose tissue and resident muscle macrophages) throughout the body (extensively reviewed previously in [11,20,26,27,28]) (Figure 3).

### 2.2. TREM2 Ligands and Signaling Cascade

**Figure 4:**
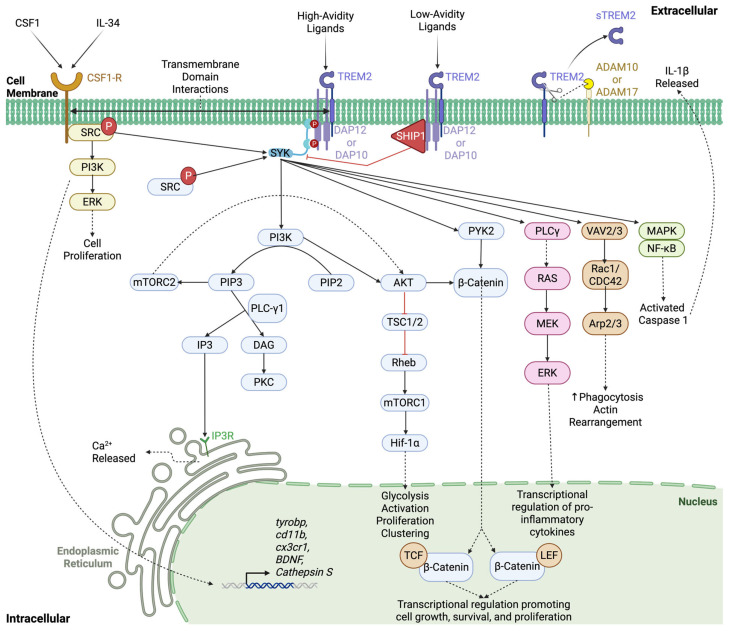
TREM2 Signaling Cascade. Abbreviations: Colony Stimulating Factor 1 (CSF1), Interleukin-34 (IL-34), Colony Stimulating Factor 1 Receptor (CSF1-R), Src Family Kinases (SRC), phosphatidylinositol 3-kinase (PI3K), extracellular signal-regulated kinase (ERK), Triggering Receptor Expressed on Myeloid Cells 2 (TREM2), DNAX activation protein of 12kDa (DAP_12_), DNAX activation protein of 10kDa (DAP_10_), Spleen Tyrosine Kinase (SYK), phosphatidylinositol-4,5-bisphosphate (PIP2), phosphatidylinositol-3,4,5-trisphosphate (PIP3), mammalian target of rapamycin complex 2 (mTORC2), phospholipase *γ*1 (PLC-*γ*1), diacylglycerol (DAG), inositol trisphosphate (IP3), protein kinase C (PKC), inositol trisphosphate receptor (IP3R), calcium (Ca^2+^), protein kinase B (AKT), Tuberous Sclerosis Complex-1 and -2 (TSC1/2), Ras homolog enriched in brain (Rheb), mammalian target of rapamycin complex 1 (mTORC1), hypoxia-inducible factor 1-alpha (Hif-1α), Proline-rich Tyrosine Kinase 2 (PYK2), T cell factor (TCF), lymphoid enhancer-binding factor (LEF), phospholipase *γ* (PLC-*γ*), mitogen-activated protein kinase kinase (MEK), Vav Guanine Nucleotide Exchange Factor 2 and 3 (VAV2/3), Ras-related C3 botulinum toxin substrate 1 (Rac1), cell division control protein 42 homolog (CDC42), actin-related protein 2/3 (Arp2/3), Mitogen-Activated Protein Kinase (MAPK), Nuclear Factor- *ϰ*B (NF- *ϰ*B), interleukin-1β (IL-1β), soluble TREM2 (sTREM2), A Disintegrin and Metalloprotease 10 (ADAM10), A Disintegrin and Metalloprotease 17 (ADAM17), and SH2-containing inositol 5’-phosphatase-1 (SHIP1).

Numerous reviews have summarized TREM2 signaling cascades, predominantly in microglia but also in osteoclasts, Schwann cells, and peripheral macrophages. Generally, the TREM2 signaling cascade is conserved between cell types, although slight nuances may exist, primarily due to differing ligands that cell types may be exposed to. Below, we provide a comprehensive, up-to-date depiction of the TREM2 signaling cascade, complied from the following reviews and research articles [9,11,13,20,22,24,25,28,29,30,31,32,33,34,35,36,37,38,39,40,41] (Figure 4).

Several ligands—including but not limited to phospholipids (e.g., phosphatidylserine) [42,43,44,45], sulfatides, lipoproteins (e.g., Apolipoprotein E, APOE) [46,47,48], nucleic acids [49], lipopolysaccharide (LPS) [50], mycobacteria [51], amyloid-β [42,52], and TAR DNA-binding protein 43 (TDP-43) [53,54]—bind TREM2 with high or low affinity. High-avidity ligands that bind TREM2 with full affinity result in the phosphorylation of the immunoreceptor tyrosine-based activation motif (ITAM) portion of DAP_12_ by Src family kinases [14,55]. Phosphorylated DAP_12_ can then allow docking and activation of Spleen Tyrosine Kinase (SYK), which robustly propagates multiple signaling cascades, including those for phosphatidylinositol 3-kinase (PI3K), Proline-rich Tyrosine Kinase 2 (PYK2), Phospholipase *γ* (PLC*γ*), Vav Guanine Nucleotide Exchange Factor 2 and 3 (VAV2/3), Mitogen-Activated Protein Kinase (MAPK) and Nuclear Factor- Kappa B (NF- *ϰ*B), ultimately leading to increased cell survival, cytoskeletal rearrangement, proliferation, phagocytosis, and release of inflammatory chemokines [9,11,20,22,24,25,29,30,31,32,33,34,35,36,37,38,39,40,41] (Figure 4). Low-avidity ligands that bind TREM2 with partial affinity result in activation of SH2-containing inositol 5’-phosphatase-1 (SHIP1), which inhibits SYK and downstream signaling cascades [11,14,40]. TREM2 signaling can also be modulated by other receptors, such as Colony-Stimulating Factor 1 Receptor (CSF1-R), that bind ligands CSF1 and Interleukin-34 (IL-34). Upon activation, CSF1-R phosphorylates SRC [56], thereby activating PI3K/ERK signaling cascades that promote cell proliferation and increased transcription of several downstream signaling molecules, including *Tyrobp* (DAP_12_) [9,57,58,59,60,61]. In addition to transcriptional changes, CSF1-R and TREM2 are frequently found close to each other and can interact either through DAP_12_-SRC signaling or physically by their transmembrane domains [39,44,60] (Figure 4). DAP_12_ and SYK are utilized in numerous other signaling cascades not discussed here, that further increase the complexity of immune cell signaling. Collectively, the literature suggests that TREM2 is just one modulatory receptor in a complex web that varies depending on cell type and microenvironment.

### 2.3. sTREM2

Soluble TREM2 (sTREM2) is a truncated TREM2 variant comprising the receptor domain and a fragment of the stalk [13]. The sTREM2 protein variant can result either from cleavage of the full-length TREM2 or from secretion of alternative splice variants [13,23,62]. A Disintegrin and Metalloprotease 10 and/or 17 (ADAM10 and/or ADAM17) have been shown to cleave full-length TREM2 at the H157-S158 bond in the stalk, which allows shedding of the ectodomain, sTREM, into the microenvironment [11,13,23,24,62,63,64,65] (Figure 4). The remaining transmembrane domain can be further cleaved by *γ*-secretase, releasing the intracellular domain for proteolysis [23,64]. Two alternative splice isoforms of TREM2, which lack the transmembrane domain, can also be translated into sTREM2, which is subsequently released extracellularly [19,66,67,68]. Once released by either mechanism, sTREM2 can be detected in the extracellular space, cerebrospinal fluid, and serum [11,13,69,70]. sTREM2 has been extensively studied in Alzheimer’s disease (AD) but has also been found in other motor/spinal neuropathologies such as ALS and following spinal cord injury [70]. Although the exact mechanisms and function of sTREM2 remain to be fully elucidated, it has been found to function independently from TREM2 by: (1) interacting with neighboring cells in a non-cell autonomous manner, (2) autocrine signaling, and (3) scavenging excess TREM2 ligands [11,13,49,63,66,71,72,73]. In AD, sTREM2 is proposed to perpetuate neuroinflammation, promote immune cell survival, and increase phagocytosis of protein aggregates, debris, and damaged neurons, as the level of sTREM2 in cerebrospinal fluid and serum correlates with disease progression [40,74,75,76]. Alternatively, sTREM2 may be useful as a biomarker for neurodegeneration following injury or disease. Individuals with ALS have been shown to have elevated TREM2 protein in the spinal cord and significantly higher sTREM2 in cerebrospinal fluid and serum compared to healthy controls [70,77]. Furthermore, cerebrospinal fluid sTREM2 levels have been shown to predict patient survival duration [78]. In spinal cord injury, elevated cerebrospinal fluid sTREM2 has also been identified and correlated with neurodegeneration markers (e.g., neurofilament light chain) within the first two months post-injury, suggesting sTREM2 may be a biomarker for injury severity [79].

### 2.4. TREM2/DAP12 Mutations in Disease

Some *TREM2* mutations have been identified that alter function and can be either disease-causing or risk factors for certain diseases. Polycystic Lipomembranous Osteodysplasia with Sclerosing Leukoencephalopathy (PLOSL), also known as Nasu–Hakola disease, is caused by recessive inheritance of homozygous loss-of-function mutations of TREM2 or DAP_12_ [80,81,82]. This disease is characterized by cysts developing on the bones as well as a presenile dementia with progression similar to Frontotemporal Dementia (FTD) [82]. Homozygous point mutations associated with the development of PLOSL include the Y38C, T66M, and V126G mutations, among others (Table 1) [83]. While the link of TREM2/DAP_12_ signaling to PLOSL is well established, the exact disease mechanisms have yet to be elucidated due to the inability of mouse models to recapitulate the disease fully.

Loss-of-function TREM2 mutations can also cause FTD-like dementia without the presence of bone cysts [84]. Mutations in exon 2 of *TREM2* are frequently found in individuals with FTD. R47H, Q33X, S116C, H157Y, and T66M mutations, among others, have been linked to FTD development (Table 1) [85,86]. Many of these mutations, especially R47H, also occur in individuals who develop AD.

The role of TREM2 variants in other disease models is less clear and often highly debated. The R47H TREM2 mutation substitutes an arginine for a histidine near the ligand-binding site and was discovered to be a risk factor for developing AD [84,87]. This discovery was the first to directly connect TREM2 dysfunction to AD development later in life. TREM2’s role in AD has been studied and described in depth by many groups [84,87,88,89,90,91]. Briefly, the connection between TREM2 function and AD is supported by showing that *Trem2* gene deletions increase Tau interneuronal dispersion [88], decrease amyloid-β plaque clearance [92], and worsen disease prognosis in mouse models. Similarly, the R47H variant has reduced affinity for amyloid-β, and this variant may hasten AD progression due to an increased affinity for externalized phosphatidylserine (ePtdSer), an “eat me” signal that can prompt synapse removal and worsen neurodegeneration [89].

Kober et al. (2016) investigated structural changes to TREM2 that occur due to various mutations using X-ray crystallography and analysis [83]. They found that AD-linked mutations tend to affect the external, ligand-binding areas of the protein, whereas other mutations may affect the internal structure of the TREM2 protein, and therefore may affect the stability and potentially impair trafficking of the protein to the cell membrane (Table 1) [83]. While each mutation likely leads to some sort of loss-of-function, it seems that disease progression and severity associated with TREM2 mutants vary greatly along a gradient of functionality. The exact mechanisms explaining how these variations lead to different neurodegenerative disorders have yet to be fully elucidated.

TREM2 mutations have been investigated in conjunction with other neurodegenerative diseases as well. Further studies extended the role of the R47H mutation to other neurodegenerative disorders, including PD [93]. However, when this mutation was investigated in the spinal cord as a potential risk factor for ALS, the results varied. Some studies proposed the R47H mutation as a risk factor in sporadic ALS [94]. However, other groups found no significant relationship between this genetic mutation and sporadic ALS in human patient populations [90]. Other *TREM2 gene* mutations in ALS have also been studied, but their potential as risk factors remains debated [90,95].

**Table 1:** Identified TREM2 mutations and associated pathology. Abbreviations: PLOSL (Polycystic Lipomembranous Osteodysplasia with Sclerosing Leukoencephalopathy, also known as Nasu–Hakola disease), AD (Alzheimer’s disease), FTD (Frontotemporal Dementia), ALS (Amyotrophic Lateral Sclerosis), and PD (Parkinson’s disease).

Mutation	Region	Associated Pathology	Reference
E14X	Signal Peptide	PLOSL	[81]
Q33X	Ig Domain	AD, PLOSL, FTD	[84,85,96,97,98,99,100,101]
Y38C	Ig Domain	AD, PLOSL, FTD	[83,84,97,101]
W44X	Ig Domain	PLOSL	[102]
R47H	Ig Domain	AD, ALS, FTD, PD	[84,85,93,94,103]
W50C	Ig Domain	PLOSL	[104]
R62H	Ig Domain	AD, ALS	[84,95,96,105]
T66M	Ig Domain	AD, PLOSL, FTD	[83,84,85,101,106,107]
N68K	Ig Domain	AD	[84]
W78X	Ig Domain	PLOSL	[80]
D86V	Ig Domain	FTD	[108]
D87N	Ig Domain	AD, ALS	[84,95]
T96K	Ig Domain	AD, FTD	[84,103,109]
R98W	Ig Domain	AD	[84]
S116C	Ig Domain	FTD	[85]
V126G	Ig Domain	PLOSL	[83,99]
D134G	Ig Domain	PLOSL	[80]
R136Q	Stalk	AD	[84]
H157Y	Stalk	AD, FTD	[84,86,110]
K186N	Helix	PLOSL	[80]
W198X	Tail	FTD	[107]
L211P	Tail	AD, FTD	[84,109,111]

### 2.5. Different Mouse Models May Contribute to Conflicting TREM2 Results

One problem is that TREM2’s functional complexity throughout development and after disease onset may induce confounding effects. The most used global *Trem2* knockout (GKO) mouse model has a 175bp deletion and introduces an early stop codon after bp17, leaving no functional protein isoform (Jax #027197) [112]. The benefit of this model is the complete absence of TREM2 signaling. However, TREM2’s actions in normal developmental synaptic pruning introduce confounds when studies look at aberrant circuitry caused by injury or disease [113]. Moreover, these results could be region-specific [114,115,116], and the unknown actions in other brain cells and the peripheral immune system can introduce pleiotropic effects that are difficult to control. Further, *Trem2* GKO can lead to conflicting conclusions depending on the neurodegenerative mouse model. For example, Leyns et al. (2017) found that in a pure tauopathy model of AD, *Trem2* knockout was beneficial and neuroprotective [117]. Two years later, the same group, in Leyns et al. (2019), found *Trem2* knockout and the R47H variant to be detrimental in an APPPS1–21 mouse model of AD [118]. This highlights the importance of accounting for the nuances of TREM2 signaling in various mouse models of disease.

In contrast, conditional *Trem2* knockout (cKO) mouse models have the benefit of being cell-type specific and potentially temporally controlled. This model usually involves a floxed mouse model that removes exons 2 and 3 from the *Trem2* gene after recombination (Jax #029853) [119]. However, there are some studies suggesting that a *Trem2* variant can still be generated without these exons [21]. The extent of signaling that can occur without the presence of exon 2, where the ligand-binding domain is located, has yet to be fully characterized; however, this TREM2 isoform variant could still fold and localize normally even without exons 2 and 3 [21].

## 3. The Dorsal Root Ganglion

### 3.1. Anatomical Overview of the Dorsal Root Ganglion

The Dorsal Root Ganglion (DRG) is a complex structure situated bilaterally within the dural sheath in the vertebral foramina, outside of the spinal cord [120]. As an enlargement of the dorsal root, a single DRG houses the cell bodies of pseudo-unipolar sensory neurons, ranging from 15,000 neurons in rats to 70,000 in humans, with variability depending on the spinal segment [121,122,123,124]. The bifurcating axons of DRG neurons extend peripherally to innervate sensory receptors or terminate freely in skin, muscle, and viscera, whereas the central branches enter the spinal cord, allowing information from the periphery to reach the CNS. Given the peculiar anatomy, DRG sensory neurons and their axons interact with many different cell types and tissues, which include myelinating or non-myelinating Schwann cells in peripheral nerves and oligodendrocytes centrally, while the cell body is surrounded by satellite glial cells. In addition, fibroblasts, pericytes, mast cells, endothelial cells, resident macrophages, and monocyte-derived macrophages can also be found among the connective tissue within the DRG and in peripheral nerves, while microglia interact with the central branches of sensory axons [125]. Following injury or in diseases such as diabetic neuropathy, communication from the periphery to the CNS is compromised [126,127]. Therefore, identifying mechanisms that can preserve, prevent, or prolong detriments is crucial for improving patient prognosis in injury and disease. 

### 3.2. TREM2 in the Dorsal Root Ganglion

Numerous RNA sequencing studies have found macrophages with high levels of *Trem2* and *Tyrobp* expression in the DRG following injury or in diseased states, but the exact role of these TREM2/DAP_12_+ cells is currently unknown [126,128,129,130]. Some of the literature suggests that macrophages in the DRG are specifically involved in the initiation and maintenance of mechanical hypersensitivity following peripheral nerve injury, as DRG macrophage depletion prevents and/or stops neuropathic pain [131]. In 2023, Feng et al. identified four different populations of macrophages in the DRG following peripheral nerve injury: (1) bone-marrow-derived precursor cells, (2) self-renewing macrophages, (3) microglia-like macrophages, and (4) satellite glial cell-like macrophages [132]. The potential differential expression and signaling of TREM2 in different DRG macrophage subpopulations remains to be elucidated. Research on the role of TREM2 in other diseases and injuries suggests that TREM2+ DRG macrophages may regulate the local cytokine response (either pro- or anti-inflammatory) and influence the metabolic processes of neighboring cells; however, TREM2 in the DRG is currently understudied (Figure 1).

## 4. TREM2 in the Spinal Cord

### Anatomical Overview of the Spinal Cord

The spinal cord contains sensorimotor circuits that can act independently from the brain (reflexes) or transmit sensory, motor, or autonomic signals between the brain and peripheral targets. The spinal cord can be divided into five regions, each with different segments that innervate different regions of the body via bilateral peripheral spinal nerve pairs. Humans have 31 segments: cervical (C1–C8), thoracic (T1–T12), lumbar (L1–L5), sacral (S1–S5), and 1 coccygeal; these vary slightly in different species (mouse: 8 cervical, 13 thoracic, 6 lumbar, 4 sacral, and 3 coccygeal). Spinal cord segments contain specific circuitry that controls different body regions and functions. For example, all segments are capable of transmitting sensory information from cutaneous organs informing about touch (mechanoreceptors), pain (nociception), or from muscles and joints (proprioception). All segments contain somatic motor neurons whose outputs control muscle contractions in the corresponding segments. Similarly, all segments have circuitry to evoke nociceptive flexion reflexes (i.e., withdrawal reflex) and proprioceptive reflexes (i.e., stretch reflex). Other circuits and functions are more specific to certain segments. The cervical and upper thoracic spinal cord contains circuitry for tectospinal reflexes coordinating eye and neck muscles, as well as respiratory reflexes that control diaphragm and intercostal muscles. Thoracic and upper lumbar regions contain circuitry that controls the autonomic sympathetic system and encodes visceral reflexes for cardiovascular control. The sacral and coccygeal regions predominantly contain reflexive circuitry for the pelvic area, including urination, defecation, and reproductive organs controlled by specialized somatic motor neurons and autonomic parasympathetic neurons [133,134,135,136,137,138]. Disease or injury may differentially affect specific spinal cord segments, causing a multiplicity of different combinations of sensory, motor, or visceral dysfunctions and symptoms.

**Figure 5:**
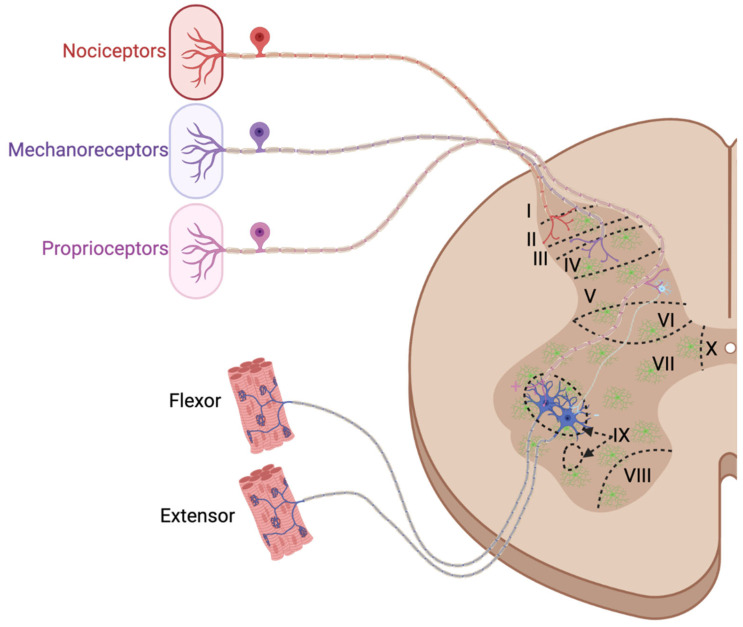
Simplified illustration of spinal reflex circuitry. The figure illustrates the different types of sensory neurons and where they terminate within a lumbar spinal cord segment. Nociceptors (red) terminate on interneurons (not shown) in lamina I-II, mechanoreceptors (purple) terminate on interneurons (not shown) in lamina III-IV, and proprioceptors (pink) terminate on interneurons in lamina V (light blue) and motoneurons in lamina IX (dark blue). Microglia (green), the spinal cord’s resident immune cell, are predominantly responsible for synapse and neuron removal in healthy development, aging, disease, and injury.

Although functional and cellular differences exist between spinal segments, all have the same general anatomy. The central projection of sensory neuron afferents enters the dorsal horn of the spinal cord through the dorsal root. Nociceptive sensory neurons terminate on interneurons in lamina I-II; mechanoreceptive sensory neurons terminate on interneurons in lamina III-V; and proprioceptive sensory neurons terminate either on interneurons in laminae V and VII or on motoneurons in lamina IX. Motoneuron cell bodies are located in the ventral horn, and their axons exit through the ventral root [133] (Figure 5). Sensorimotor integration function in the spinal cord occurs through reflex circuits that commonly experience plasticity during development, aging, disease, and after injury. The removal of synapses or damaged neurons during aging, disease, and injury is frequently attributed to microglia. Microglia are the primary spinal cord cells that contain TREM2; however, in diseased or injured states, TREM2+ peripheral macrophages may enter the spinal cord as well. In this section, we will discuss the role of TREM2 in microglia function broadly, followed by the implications of TREM2 in the dorsal and ventral horns, and in spinal cord injury.

## 5. TREM2 in Microglia

### 5.1. Disease-Associated Microglia

High TREM2 expression is frequently associated with a subtype of activated microglia known as Disease-Associated Microglia (DAM). Morphologically, DAM typically have enlarged cell bodies and reduced processes, resulting in an ameboid appearance compared to ramified surveying microglia in healthy tissue [139,140,141,142]. DAM were first characterized in AD mouse models and human post-mortem tissue, as they are found around amyloid-β plaques and in proximity to apoptotic neurons. However, DAM have also been found in ALS, PD, peripheral nerve injury, spinal cord injury, and aging rodent models [142,143,144,145,146]. Through RNA sequencing and immunohistochemistry in various neuropathologies, DAM have been characterized by elevated expression of *Trem2*, *Tyrobp*, *Apoe*, *Lpl* (Lipoprotein Lipase), and *Ctsd* (Cathepsin D), cumulatively deeming their typical function as pro-inflammatory, lipid metabolizing, and highly phagocytic [144,145,146,147,148,149,150]. The formation of DAM from surveying microglia has been generally accepted to be a two-stage process: Stage 1 is TREM2-independent, and Stage 2 is TREM2-dependent. Stage 1 involves the downregulation of homeostatic genes (e.g., *Cx3cr1* and *Tmem119*) as well as the upregulation of *Trem2*, *Tyrobp*, and *Apoe*. TREM2-dependent Stage 2 results in expression changes in genes involved in phagocytosis and lipid metabolism (e.g., *Lpl* and *Ctsd*) [144,145,147,148]. Research suggests that increased in APOE expression helps sustain TREM2 activation in an autocrine/paracrine loop fashion [151]. Further, as discussed above, TREM2 activity results in pro-proliferative and pro-survival signaling cascades, which maintain microgliosis in diseased or injured states [148]. Genetic *Trem2* knockout or xenotransplanted human knockout microglia in AD mouse models (5XFAD), and *Trem2* knockout in ALS mouse models (SOD1) all result in fewer DAM [91,146,152]. Similarly, knockout of SYK, a downstream target of activated TREM2, also results in reduced phagocytosis and DAM formation [153]. In contrast, we recently reported that microglial *Trem2* knockout mice still develop what appear to be DAM following peripheral nerve injury, although their microglial function is compromised [142]. Collectively, this highlights: (1) the necessity to investigate microglial TREM2 in additional disease and injury models, (2) the variability of TREM2 function depending on each cell’s microenvironment, and (3) the possibility that DAM formation may occur through compensatory pathways activated in certain disease or injury states.

### 5.2. Synaptic Plasticity

TREM2 has also been shown to be involved in synaptic plasticity; however, its exact role in synapse removal is complex [154]. Filipello et al. (2018) demonstrated that TREM2 is required for the canonical synaptic pruning that occurs during developmental circuit and behavior refinement [113]. Juvenile *Trem2* knockout mice result in increased cortical synaptic density, excess excitatory neurotransmission, reduced functional brain connectivity, and ultimately social behavior deficits. However, the lack of synaptic pruning may contribute to reduced synaptic stabilization, as adult *Trem2* knockout mice had reduced, rather than increased, synaptic density [113]. In agreement, Jay et al. (2019) found that *Trem2* knockout resulted in decreased synaptic density in one-month-old mice; however, they mechanistically proposed that microglia TREM2 limits astrocytic synapse engulfment during development [155]. Intriguingly, post-mortem cortex from 5- to 23-year-old idiopathic autistic humans had reduced TREM2 compared to control tissue, and TREM2 levels inversely correlated with the individuals’ Autism Diagnosis Interview-Revised (ADI-R) score, suggesting microglial TREM2 is necessary for normal canonical neuronal circuit development [113]. Recently, Vecchiarelli et al. (2024) published evidence that a particular TREM2-dependent microglia phenotype, frequently missed by immunohistochemistry, “Dark Microglia,” arises at crucial developmental periods and is involved in synaptic and vascular remodeling [156,157].

Additional studies reveal further complexities of TREM2 involvement with synaptic plasticity. During development, microglia often utilize C1q and the complement cascade to selectively prune synapses [158,159,160]. The signal to initiate complement-mediated synapse pruning was proposed to be initiated by TREM2 recognition of externalized phosphatidylserine (ePtdSer) at synaptic sites [161]. PtdSer is normally located on the inner leaflet of the lipid bilayer, but in disease and injury states, PtdSer can be externalized. ePtdSer is a known “eat me” signal and high-affinity ligand of TREM2 [43]. Nevertheless, a built-in TREM2 feedback mechanism also seems to be at play, negatively modulating synapse pruning. In AD rodent models, amyloid-β and tau also result in increased C1q, complement pathway activation, and synapse elimination [162,163,164]. Zhong et al. (2023) found microglial TREM2 binds C1q with high affinity in 5xFAD mice and postmortem human AD cortex to minimize complement-mediated synaptic loss [165]. Thus, TREM2 knockout resulted in accelerated synapse elimination, suggesting that TREM2 prevents synapse loss in AD. However, Rueda-Carrasco et al. (2023) provided evidence that in the early stage of an AD mouse (hAPP NF-L knock-in mouse), hyperactive synapses display ePtdSer and are preferentially removed by microglia via TREM2 [166]. The authors conclude that TREM2 is beneficial by selectively removing inefficient synapses. A similar conclusion was reached by Fracassi et al. (2023), who reported that non-demented individuals with AD neuropathology are protected by higher-than-normal TREM2 activity detecting ePtdSer in dysfunctional synapses, triggering their removal by microglia [167]. The discrepancy between these various studies may be explained by the use of different models and analyses at different disease stages, but all highlight that TREM2 has complex activities in synaptic plasticity, interfering with multiple mechanisms of synapse deletion/preservation. Dejanovic et al. (2022) highlight differential effects of TREM2 on microglia versus astrocytic engulfment of inhibitory versus excitatory synapses in various models of AD, creating synaptic imbalances affecting network function [168]. Moreover, TREM2 actions on synapses might be regionally dependent. TREM2-R47H variant results in increased synapses in the cortex but not in the hippocampal regions [169]. Thus, synapse deletion might occur through a multiplicity of mechanisms in different disease states, synapses, and regions, with TREM2 affecting their balance and having differential effects, sometimes beneficial, sometimes detrimental. The role of TREM2 in spinal cord synaptic plasticity in disease or following an injury has been much less studied. Freria et al. (2024) recently reported specific deletion of inhibitory synapses controlling sympathetic outflow by a TREM2-microglia mechanism after spinal cord injury, contributing to autonomic dysreflexia [170].

### 5.3. Neuronal Bioenergetic Support

Microglia have been shown to routinely monitor and rapidly respond to neuronal mitochondrial states in health and following injury via specialized microglial P2RY12-neuronal KV2.1 junctions [171]. However, the literature also suggests that alternative microglial receptors may influence microglia’s aid in neuronal bioenergetic support. In 2019, Tagliatti et al. provided novel evidence that microglial TREM2 is necessary for hippocampal CA1 pyramidal neuron bioenergetics. In this study, *Trem2* knockout postnatal day 1 (p1) mice exhibited reduced hippocampal basal mitochondrial metabolism. Excitatory pyramidal and granular cells specifically resulted in altered gene expression for mTOR and mitochondrial pathways (including mitochondrial machinery and oxidative phosphorylation components) and delayed maturation compared to wild-type littermates. Further, as expected, TREM2 knockout resulted in altered synaptic pruning. Intriguingly, the effect of *Trem2* knockout was not universal, as neither CA3 excitatory neurons nor inhibitory neurons were significantly affected. CA1 was also found to have significantly higher amounts of TREM2 than CA3 in wild-type mice. This work suggests that some neuronal subpopulations or neurons in certain states may be more dependent on microglial TREM2 than others, and that microglial TREM2 is necessary for the mitochondrial metabolism of normally developing neurons [114]. Currently, it is largely unknown how microglial TREM2 affects neuronal bioenergetics in spinal neurons or motoneurons during disease or after injury, where there is a high metabolic demand for survival. Our studies have suggested that in the absence of microglial TREM2, there is a dysregulation in motoneurons’ normal cell body reaction to axotomy and in preparing a switch towards a regenerative metabolism (see below).

## 6. TREM2 in the Dorsal Horn

### Neuropathic Pain

Physiological pain is crucial for detecting and responding to noxious stimuli. As discussed above, the flexion withdrawal reflex depends on cutaneous nociceptors to detect a painful stimulus and rapidly withdraw that part of the body from a potentially damaging stimulus. However, if pain persists post-healing or during disease, it becomes pathological. Identifying mechanisms to remedy pain sensation without compromising healing is essential for therapeutic advancement. In this section, we will discuss TREM2’s involvement in microglia function in various neuropathic pain conditions.

Following a peripheral nerve injury, neuropathic pain frequently persists in addition to permanent motor and proprioceptive deficits [142]. It is generally accepted that dorsal horn microglia contribute to plasticity, which facilitates prolonged hypersensitivity following peripheral nerve injury [172,173,174,175,176,177,178,179,180,181,182,183,184,185]. We have previously reviewed the mechanisms of microglia activation and general hypotheses behind microgliosis around the central branches of sensory afferents injured in the peripheral nerve and therefore will not be discussing them in detail here [9]. Given that microglia contribute to neuropathic pain, identifying mechanisms that alter microgliosis may provide options as therapeutic targets. Microglial TREM2 has increasingly been investigated as a mechanism of microglia-induced allodynia following peripheral nerve injury.

Numerous studies in rodent models have reported an increase in dorsal horn microglial TREM2 and DAP_12_ expression and phosphorylation following peripheral nerve injury [60,186,187,188]. In 2016, two publications provided evidence that either TREM2 or DAP_12_ deficiency can reduce allodynia following peripheral nerve injury. Guan et al. (2016) provided RNA sequencing evidence of increased dorsal horn *Tyrobp* (DAP_12_) expression as early as one day post-sciatic nerve cut-ligation [60]. In rats, animals that performed pain-induced self-mutilation of the injured limb (autotomy) had higher DAP_12_ expression than littermates with no evidence of autotomy, suggesting that allodynia may be mediated by DAP_12_ signaling. This was confirmed as DAP_12_-deficient mice had reduced allodynia, evident by increased mechanical thresholds for pain. Later that year, Kobayashi et al. (2016) confirmed these findings and showed that following an L4 nerve injury, dorsal horn microglia increased TREM2 and DAP_12_ expression and activity, evident by increased DAP_12_ phosphorylation [186]. The increase in TREM2/DAP_12_ activity resulted in an increased production of pro-inflammatory cytokines and purinergic receptors (TNFα, IL-1β, IL-6, P2RX4), ultimately leading to increased pain post-injury. Again, DAP_12_-deficient mice exhibited decreased inflammation and allodynia with improved recovery time. Perhaps the most convincing of direct TREM2 involvement in the regulation of sensory nociceptive thresholds was the intrathecal injection of a TREM2 agonist-induced allodynia via DAP_12_ activity [186]. Wang et al. (2022) further elucidated the role of TREM2-mediated allodynia in a spared nerve injury rat model [187]. Intrathecal injection of TREM2 lentivirus or an autophagy inhibitor (3-MA) both led to increased mechanical allodynia. A TREM2-autophagy pathway modulates microglia function and neuroinflammation following peripheral nerve injury. The expansion of the RNA sequencing literature has supported the notion of dorsal horn microglial heterogeneity. Recently, a specific cluster of microglia, identified as CD11c+, was found to appear following peripheral nerve injury and deemed necessary for spontaneous allodynia recovery, as CD11c+ microglia depletion caused relapse of injury-induced pain sensitivity. This subpopulation of microglia was theorized to be involved in removing myelin from damaged primary afferents. Intriguingly, mice with TREM2 deficiency have impaired appearance of CD11c+ microglia [189,190]. In AD rodent models, CD11c was found to be another marker of DAM [144,191]. Based on this, it is suggested that the identified CD11c+ microglia are TREM2-dependent DAM that contribute to allodynia post-peripheral nerve injury. Collectively, this cumulative evidence suggests that peripheral nerve injury increases microglial TREM2 signaling, thus contributing to neuropathic pain states and a decrease in sensory thresholds to cutaneous stimuli.

TREM2 influence on spinal microglia function may be more specific to females. In a recent study using a mouse sciatic nerve chronic constriction injury model, ipsilateral spinal microglia RNA sequencing data were submitted to QIAGEN’s Ingenuity Pathway Analysis to help identify the physiological function and upstream regulators of transcriptional changes between males and females seven days post-injury. Identified male microglia upstream regulators included activation of Interferon Regulatory Factor 3 and 7 (IRF3/IRF7), whereas female microglia included activation of TREM2 and Interferon-*γ*. The authors suggested sexual dimorphisms in microglial transcriptional signatures, which may contribute to differences in pain processing and thresholds post–peripheral nerve injury [192]. Recently, we showed evidence suggesting that ventral horn DAM-like microglia in females have elevated TREM2 compared to males, with sex-specific TREM2 knock-out effects, supporting the notion that female microglia may utilize TREM2 activity more than male microglia [142].

Neuropathic pain is also frequently a comorbidity of other pathologies, such as diabetic neuropathy. Type I diabetes mellitus can be modeled in rodents via intraperitoneal administration of Streptozotocin (STZ), which results in painful diabetic neuropathy, evident by spinal cord neuroinflammation, axon degeneration, and reduced mechanical withdrawal and thermal withdrawal thresholds. Chen et al. (2021) identified a marked increase in TREM2 and DAP_12_ expression between one and two weeks post-Streptozotocin injection [39]. Overexpression of TREM2 via intrathecal infusion of a microglia-specific lentivirus resulted in increased microglial number, CD68 (indicating increased phagocytosis), increased production of pro-inflammatory IL-1β, and reduced expression of anti-inflammatory TGF-β and IL-10. Blocking TREM2 activity via intrathecal injection of a TREM2-neutralizing antibody resulted in the opposite effects [193]. This work supports the theory that dorsal horn TREM2 activity contributes to neuroinflammation-mediated chronic pain in diabetic neuropathy models, similar to post-peripheral nerve injury and neurodegenerative diseases.

Bone fractures and orthopedic surgery resulting in neuropathic pain are resistant to current analgesics, which further warrants research to identify pharmacological targets for pain relief. An anti-malaria drug, Artesunate, was proposed as a potential pain-relief treatment due to its anti-inflammatory properties [194,195]; however, if and how Artesunate might aid in neuropathic pain from fractures and orthopedic surgery was unknown. Zhang et al. (2022) reported that intrathecal injection of Artesunate four to six days following a bone fracture ameliorated neuropathic pain by inhibiting upregulation of CCL21, TREM2, and DAP_12,_ resulting in reduced allodynia and improved mechanical and thermal thresholds [196]. Other research has shown that Artesunate treatment in the periphery following peripheral nerve injury also improves nerve regeneration and reduces inflammation, although it is unknown if the effects are also TREM2-mediated [197].

Chemotherapy is known to induce peripheral neuropathy and microglia-mediated neuropathic pain [198,199,200,201,202]. The chemotherapy agent cisplatin was found to induce prolonged microgliosis in Lumbar 4–6 dorsal horn and increase microglial TREM2 and DAP_12_ expression. Strikingly, intrathecal injection of an anti-TREM2 neutralizing antibody 30 min post-cisplatin administration resulted in reduced pro-inflammatory cytokines (IL-6, TNFα, iNOS), increased anti-inflammatory cytokines (IL-4, IL-10), reduced allodynia, and reduced the loss of small nerve fibers in the periphery. This suggests that cisplatin-induced neuropathic pain may be mediated by TREM2/DAP_12_ signaling [199].

**Figure 6:**
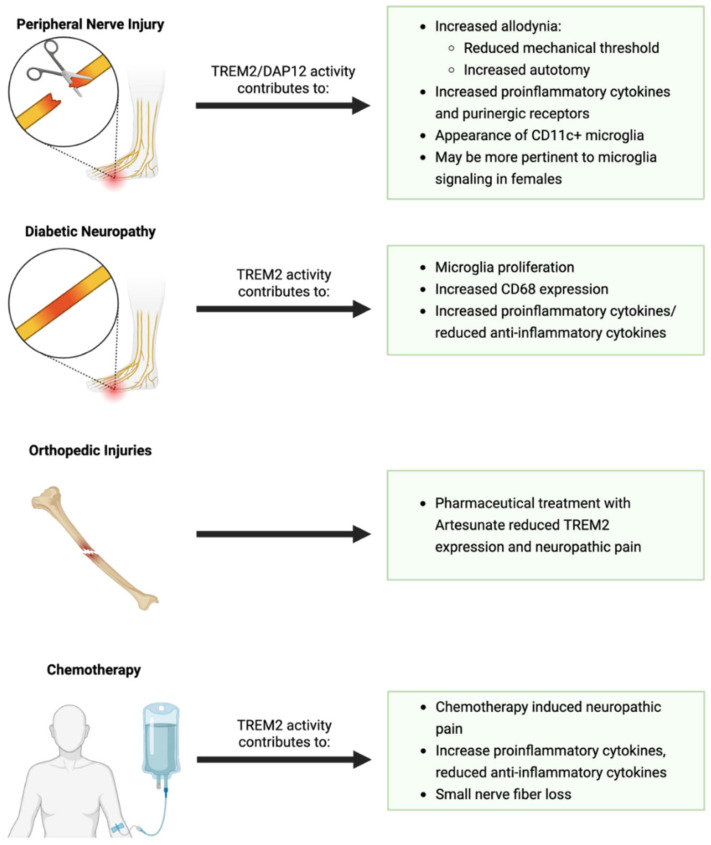
TREM2 contribution to dorsal horn inflammation and allodynia in four neuropathic pain conditions.

Collectively, the literature suggests dorsal horn TREM2/DAP_12_ activity contributes to neuropathic pain regardless of injury type and thus suggests it as a potential therapeutic target for analgesic drug development (Figure 1 and Figure 6).

## 7. TREM2 in the Ventral Horn

### 7.1. Peripheral Nerve Injury

Microglia are known to have complex interactions with healthy and injured motoneuron cell bodies and dendrites located in the ventral horn [9,142,203,204,205,206,207]. Following a peripheral nerve injury, microglia become activated, proliferate, migrate, and adhere to motoneuron cell bodies, displaying a heterogeneity of microglia morphological phenotypes [142,206]. TREM2 and DAP_12_ expression, as well as phosphorylation of downstream SYK, increase in microglia near cell bodies of axotomized motoneurons [142,208,209]. Within activated microglia, those expressing the highest levels of TREM2 also display elevated phagocytic markers, like CD68, compared to neighboring microglia [142,208]. Following hypoglossal nerve injury in DAP_12_-deficient mice, microglia had reduced expression of pro-inflammatory cytokines and there was reduced motoneuron cell death, suggesting a neuroprotective role for TREM2 [209]. In support of this, we recently provided evidence in global and conditional TREM2 knockout mice that microglial TREM2 is necessary for the canonical chromatolysis reaction that follows motor axon axotomy [142]. Chromatolysis is generally regarded as a morphological sign of regenerative capacity that parallels the exponential increase in RNA and protein synthesis required for axon regeneration [210,211]. In addition, we found TREM2 highly expressed in microglia macroclusters with typical characteristics of DAM, containing high numbers of large CD68 granules and suggesting macrophagocytic activity. These microglia clusters are associated with degenerating motoneurons and attract infiltrating CD8+ T cells [206]. TREM2 deletion did not alter the formation of these clusters but decreased the expression of phagocytic markers. Collectively, these results suggest that TREM2 activity in the ventral horn after peripheral nerve injuries has a dual function: first, it may facilitate the regenerative phenotype of some motoneurons; and second, it may promote cell death and/or clearance of other motoneurons.

### 7.2. Aging

Aging gradually increases microglial activation, phagocytosis, and contacts with the cell bodies and excitatory synapses of α-motoneurons in the ventral horn of mice [212,213]. These findings suggest that microglia may play a role in either motoneuron maintenance with age or in their decline, resulting in loss of motor force and increased fatigability. RNA sequencing of microglia from aged mice shows an increase in pro-inflammatory and DAM markers (e.g., *Spp1*, *Lpl*, *Apoe)* and indicates TREM2 as an upstream regulator of aging-related transcriptional changes [212]. Additionally, motoneurons show increased expression of APOE, a TREM2 ligand, with age [214]. These results may point to a preferential effect on motoneuron dysfunction and suggest the APOE-TREM2 pathway as a potential therapeutic target for mitigating aging-related motor decline (Figure 1).

### 7.3. Amyotrophic Lateral Sclerosis

Selective and progressive loss of motoneurons accompanied by neuroinflammation in the spinal cord is a hallmark of ALS. The literature suggests a duality of microglia function in ALS, with microglia having an anti-inflammatory neuroprotective role at early disease timepoints and gradually becoming pro-inflammatory and pro-degenerative as the disease progresses [215,216,217,218]. Notably, DAM increase with ALS disease progression in humans and mice [91,219,220,221]. Because DAM formation and function are tightly coupled to TREM2 [144,145,146,147,148,149,150], TREM2 has become a potential therapeutic target of interest for delaying ALS progression.

Post-mortem human ALS ventral horns display increased *TREM2*, *Tyrobp*, and *Apoe* mRNAs [222]. Spatial transcriptomics performed on ventral horns from the SOD1 ALS mouse model suggests increased *Trem2* and *Tyrobp* mRNA expression begins presymptomatically [223]. Further, *Trem2* knock-out SOD1 mutant ALS mice have increased homeostatic microglia (identified by *P2ry12*) and reduced pro-inflammatory microglia (identified by *Clec7a*, *Apoe*, *Csf1*) in lumbar spinal cord ventral horns [91]. These results advocate for the involvement of TREM2 early in disease pathology and a TREM2-dependent induction of a pro-inflammatory microglia state in ALS.

Other findings propose that TREM2 functions may be neuroprotective in ALS models. Mislocalization, misfolding, and aggregation of TDP-43 contribute to ALS progression [224,225,226]. In *Trem2* knockout mice with AAV-induced overexpression of human TDP-43 (hTDP-43), aggregation increased and clearance was reduced, leading to more rapid disease progression and exacerbated loss of motor function compared to wild-type controls [53]. TDP-43 is a known ligand of TREM2 [53,54]. Thus, without TREM2, microglia fail to respond to the presence of TDP-43 and to exert what appears to be a beneficial role of TREM2 in ALS pathology (Figure 1).

## 8. Spinal Cord Injury

Spinal cord injuries commonly result in incomplete damage with spared tissue, allowing some transmission of signals and partial recovery of function [227,228,229,230]. Whether lesions are complete or include spared tissue, there are transcriptional and neuronal circuitry changes outside the lesion zone that contribute to broader effects not strictly localized to the lesion zone or a particular spinal cord area. Another important property is the site of the lesion (cervical, thoracic, lumbar), each impairing different regions of the body. Moreover, spinal cord injuries can result from contusions (“bruising” from blunt force trauma), compression (prolonged pressure on the spinal cord), distension (stretching of the spinal cord), dislocation (shifting of the spinal cord due to dislocated vertebrae), or laceration/transection (cutting of the spinal cord) [231]. Depending on the severity, type, and location of the injury, spinal cord injuries can result in different levels of sensory, motor, and autonomic disturbances. In terms of spinal reflexes involving skeletal muscle specifically, spinal shock occurs immediately following injury, where reflexive circuitry below the injury is temporarily lost or diminished [232,233,234]. The greater the severity of the injury, the more pronounced and the longer the duration of the spinal shock. Over time, reflexive circuitry in segments below the injury often returns, frequently resulting in hyperreflexia [233].

The pathophysiology of spinal cord injury is two-part, where the physical damage results in the primary injury, which may be followed by a secondary damage resulting from neuroinflammation, demyelination, excitotoxicity, scarring, apoptosis, as well as whole-body complications (e.g., immunosuppression, neuroendocrine dysfunction, and gastroesophageal reflux disease) [235,236,237,238,239,240]. Following spinal cord injury, microglia govern many cellular responses and thereby promote or hinder functional recovery [241,242]. Numerous reviews have covered microglia function following spinal cord injuries, which will not be repeated in detail here [241,242,243,244,245,246,247,248,249]. They suggest microglia involvement in the formation of the glial scar, the recruitment of peripheral macrophages, phagocytosis of debris, neuronal plasticity, changes to the Blood–Brain Barrier (BBB), and general tissue inflammation. We will focus on the potential roles played by microglial TREM2, which has been increasingly investigated recently.

Microglia are necessary for coordinating initial injury responses and infiltration of peripheral macrophages following mouse T9 dorsal contusion spinal cord injuries [241]. PLX5622-induced microglial depletion before spinal cord injury resulted in: (1) reduced astrocyte and NG2 cell (polydendrocyte) proliferation, which contributed to poor glial scar formation around the lesion core, (2) reduced peripheral macrophage infiltration, (3) diffusion of foamy macrophages that successfully infiltrated into spared white matter region outside of the lesion core, and (4) altered transcription and phenotypes of both infiltrating peripheral macrophages and activated astrocytes which lead to reduced inflammatory gene upregulation, lipid processing, cell adhesion, and proliferation. These changes exacerbated myelin and axon pathology and resulted in increased locomotor deficits/instability 14–35 days post-injury compared to controls. *Trem2* and *Syk* mRNA expressions were increased following spinal cord injury and were dependent on microglia activation. Specifically, *Trem2* was suggested to be involved in governing phagocytosis, endocytosis, and cytokine response, whereas *Syk* was suggested to be involved in the functions mentioned above, as well as protein secretion and cytokine production [241]. This work led Zhao et al. (2025) to investigate the role of TREM2 specifically, after a T9 dorsal contusion injury model [250]. *Trem2* expression increases from one to seven days post-injury and is positively correlated with genes that regulate lysosome membrane permeabilization and phagocytosis, including *Cd68.* In wild-type animals, TREM2 is frequently found near the lesion border, similarly to LAMP1 (Lysosome Associated Membrane Protein 1), a marker of autophagy. Surprisingly, RNA sequencing suggested improved autophagy and lysosomal pathways in *Trem2* GKO mice compared to wild-type mice following spinal cord injury [250]. By silencing *Trem2* in BV2 cells (a murine microglia cell line) with an siRNA in vitro, the authors suggest that TREM2 improves microglial autophagy by reducing lysosome membrane permeability and thus improving microglia mitochondrial metabolism. These results were confirmed in vivo in *Trem2* GKO mice seven days post-injury [250]. Zhao et al. (2025) confirmed Brennan et al. (2022)’s results that *Trem2* GKO reduces glial scar formation [241,250]. However, contrary to Brennan et al.’s microglia depletion results, which caused exacerbated locomotor deficits, Zhao et al. [250] found that TREM2-deficient mice demonstrated improved hindlimb motor evoked potential and hindlimb gait kinematics compared to wild-type mice 35 days post-injury. This may suggest that microglial TREM2 may be detrimental for functional recovery. Both studies were performed in a *Trem2* GKO mouse model and, therefore, are unable to decouple potential TREM2 functions in microglia or infiltrating peripheral immune cells. Studies in microglia-specific cKO mice, or by pharmacologically altering TREM2 activity in a temporally specific manner, are necessary for distinguishing peripheral versus central and early versus delayed TREM2 involvement.

Gao et al. (2023) compared *Trem2* GKO to *Cx3cr1^CreER^;Trem2^flx/flx^* cKO mice that focused *Trem2* deletions in postnatal microglia and reported similar results as Zhao et al. (2025) [250]: both models showed improved locomotor function seven days post-injury [79]. Intriguingly, at 28 days post-injury, only GKO mice had reduced lesion size and axon degeneration. The authors suggest that this may indicate that TREM2+ infiltrating peripheral immune cells may have a greater contribution to detrimental outcomes; however, many peripheral macrophages are also CX3CR1+, meaning they also lack *Trem2* in the cKO [251,252,253]. Intriguingly, single-cell RNA sequencing of both knockout models revealed an increase in perivascular macrophages, suggesting potential alterations at the BBB. No difference in the total number of infiltrating immune cells was observed in both knockout models compared to wild-types, suggesting that TREM2 is not directly involved in peripheral immune cell recruitment. TREM2 knockout myeloid cells had reduced gene expression associated with phagocytosis and lysosomes, assessed by CD68 expression around the lesions [79]. Collectively, these results suggest that TREM2 activity may interfere with functional recovery following spinal cord injury, although improved methodology for temporal and cell-specific regulation of *Trem2* expression is needed to confirm this conclusion (Figure 1).

## 9. TREM2 Along Peripheral Axons

### 9.1. Anatomical Overview of Peripheral Nerves

Peripheral nerves contain motor, sensory, and autonomic axons in variable proportions. Withing axon fascicles multiple unmyelinated axons are surrounded by the same Schwann cell, while Schwann cells surround individual myelinated axons are. Axons and their associated Schwann cells are surrounded by an endoneurium. Multiple nerve fibers are further grouped into fascicles and surrounded by the perineurium. The epineurium further bundles multiple fascicles together to form the nerve. The vasa nervorum is the network of blood vessels that run throughout the nerve and may contain infiltrating immune cells [254,255,256,257,258]. Resident and infiltrating immune cells (including macrophages), fibroblasts, perineural cells, pericytes, and endothelial cells can all be found within the nerve as well [259,260,261,262]. Disruption of peripheral nerve function directly inhibits the transmission between the CNS and the periphery. Identification of mechanisms to promote recovery or prevent degeneration in aging, disease, and following injury is crucial for maintaining proper circuitry function, and therefore, organismal function. Below we discuss the implications of TREM2 in Schwann cells and peripheral macrophages along peripheral nerves.

### 9.2. TREM2 in Schwann Cells

Recently, Zhang et al. (2024) published novel evidence that Schwann cells express TREM2. By utilizing lentiviral [25] TREM2 knockdown in murine Schwann cell primary culture, Zhang et al. demonstrate that TREM2 deficiency results in impaired PI3K-AKT-mTOR signaling, and activated AMPK and caspases, culminating in mitochondrial damage, impaired mitochondrial metabolism/glycolytic flux, and ultimately apoptosis in Schwann cells. Furthermore, in a mouse model of Acute Motor Axonal Neuropathy (AMAN), Schwann cell TREM2 deficiency exacerbated sciatic nerve action potential conduction failures by preventing myelin debris clearance, increasing Schwann cell apoptosis, and preventing axon regeneration in vivo. Collectively, this is the first evidence of TREM2 function in Schwann cells and involvement in PNS axon regeneration (Figure 1). TREM2 axonal-Schwann cell signaling should be further explored in other disease contexts to fully understand its role in Schwann cell neuroimmune and homeostatic signaling.

Schwannomas, for example, are benign tumors formed from Schwann Cells in the CNS or PNS, but most commonly along the vestibular nerve. Around five percent of vestibular schwannomas occur due to a mutation in the *NF2* gene (which encodes Merlin protein) and is highly involved in RAS/Raf/MEK/ERK, PI3K/AKT/mTORC1, and other signaling cascades [263]. The remaining 95% of schwannoma cases are sporadic with unknown causes. The tumor microenvironment around schwannomas is diverse but highly influenced by Tumor-Associated Macrophages (TAMs), as there is a correlation between immune-suppressive TAMs and schwannoma progression [263,264]. Although TREM2 has been minimally investigated in the context of schwannomas specifically, TREM2 is a notorious marker for immune-suppressive TAMs [265,266]. In *Trem2* knockout mice with fibrosarcoma tumors, the tumor microenvironment was altered to allow increased infiltrating T cells and improved innate tumor control [265]. Fu et al. (2023) also performed computational analysis on publicly available mRNA expression datasets from individuals with sporadic vestibular schwannomas, which suggested increased TREM2 expression correlated with infiltration of immune cells in human vestibular schwannomas [267]. It is currently unknown if *TREM2* mutations are a risk factor for sporadic schwannomas in humans despite utilizing similar downstream signaling cascades, nor if TREM2 in TAMs is involved in schwannoma progression. However, the discussed results suggest TREM2 as a potential therapeutic target for peripheral schwannomas and may be translatable to other types of peripheral nerve tumors, warranting further investigation.

### 9.3. TREM2 in Peripheral Macrophages

Injury-induced axotomy of peripheral axons results in nerve fiber breakdown distal to the injury, following a process known as Wallerian Degeneration. In 1969, Olsson and Sjöstrand demonstrated that macrophages rapidly invade the nerve at the site of the injury and along the distal stump that is undergoing Wallerian Degeneration [268]. These macrophages become highly phagocytic as early as three days post-injury and progressively become “foamy” macrophages, consistent with lipid metabolism from myelin and axonal debris [262,269,270]. We now know these macrophages also utilize VEGFA-dependent mechanisms to promote angiogenesis and form vascular bridges between proximal and distal nerve stumps, allowing proliferating Schwann cells to bridge the gap and guide regenerating axons to their targets [268,271,272,273,274,275,276,277]. Within peripheral nerves, both PNS-resident macrophages and infiltrating monocyte-derived macrophages have been identified post-injury. PNS-resident macrophages are suggested to be “microglia-like” as they self-proliferate and have transcriptional profiles similar to microglia (both expressing *Tmem119*, *P2ry12*, *Siglech*, *Trem2*, and *Olfml3*), and significantly different to infiltrating monocyte-derived macrophages and DRG resident macrophages. Surprisingly, phagocytic bead assay experiments suggest surveying PNS-resident macrophages in the sciatic nerve have greater baseline phagocytic capacity than microglia in spinal cords, potentially suggesting more rapid response capabilities [261,262]. Intriguingly, following nerve injury, GO terms for PNS-resident macrophages specifically suggest involvement with angiogenesis, collagen fibril organization, nerve organization, and axon guidance [261,262]. It remains unclear whether PNS-resident macrophages are the specific subtype of cells responsible for crucial angiogenic bridging between nerve stumps. Following sciatic nerve injury, RNA sequencing experiments have revealed that foamy PNS-resident macrophages display elevated *Trem2*, *Cd68*, *Arg1*, and *App1* [262,277], suggesting involvement with lipid metabolism and an immunosuppressive response [262]. Collectively, it is plausible that PNS-resident macrophages may utilize TREM2 to rapidly detect damaged axons, engulf myelin debris, aid in initiating angiogenesis to promote nerve stump bridging, and ultimately resolve inflammatory responses over time. Based on this literature, we postulate that TREM2 in PNS macrophages may be beneficial following axotomy.

The involvement of PNS macrophages in other neuropathies is an emerging field of study. In mutant SOD1 ALS rodent models, macrophages infiltrate the peripheral nerve in the pre-symptomatic stage, become foamy, and are filled with mutant SOD1 [278,279,280,281]. This finding was confirmed in humans with sporadic ALS [282]. It is generally accepted that peripheral nerve inflammation is not the cause of ALS, but instead a response to early disease changes. However, peripheral macrophages can significantly impact disease progression, as modifying peripheral macrophages to be anti-inflammatory reduces CNS microgliosis, transcriptionally alters microglia to be more “supportive,” and extends the life span of ALS mice [282]. The role of PNS-resident macrophages along peripheral axons in the context of Guillain–Barré syndrome, chronic inflammatory demyelinating polyradiculoneuropathy, diabetic polyneuropathy, Chemotherapy-induced peripheral neuropathy, and Charcot–Marie–Tooth Disease was recently reviewed by Msheik et al. [283]. Generally, it is agreed that the microenvironment around PNS resident macrophages greatly shapes their response and function, which can make it difficult when analyzing PNS-resident macrophages in different nerves, organisms, or neuropathies. However, the finding that modulating peripheral macrophages can alter CNS microglia response and disease prognosis makes it a therapeutic target regardless of being beneficial or detrimental. Whether TREM2 in PNS-resident macrophages contributes to the heterogeneity of immune response across neuropathies is currently understudied.

## 10. TREM2 in Muscle

### 10.1. Anatomical Overview of Skeletal Muscle

Skeletal muscle not only functions to allow organismal movement and postural control, but also houses proprioceptive receptors, which provide a sense of spatial self-awareness and function to prevent muscle injury. A single muscle is covered by a connective tissue layer called the epimysium. The perimysium further divides the muscle into bundles, which allows vasculature and nerves to run through the body of the muscle [284,285]. Muscle length is detected by Ia and II sensory afferents that wrap around intrafusal muscle fibers in muscle spindles surrounded by a fibrous muscle capsule [286,287,288]. Ib sensory afferents innervate the Golgi Tendon Organs and detect muscle force. β- and *γ*-motoneuron innervation of intrafusal fibers control the sensitivity of the muscle spindle and help regulate muscle tone [289]. Extrafusal muscle fibers, located outside the muscle capsule, are innervated by α- and β-motoneurons, generally accepted to be force-generating during organismal movement [290,291]. The location at which a motoneuron synapses onto muscle fibers is called the Neuromuscular Junction (NMJ). Here, a motoneuron’s axon terminal aligns with a muscle’s postsynaptic region, characterized by multiple post-junctional folds. The motor axon releases acetylcholine, which binds to muscle nicotinic acetylcholine receptors, causing a muscle action potential that ultimately triggers muscle contraction [284,285]. Terminal Schwann cells are a non-myelinating glial cell that encases the NMJ and function to provide structural support, neurotrophic support, neurotransmission regulation, and aid in synapse remodeling during development and reinnervation following injury [292,293,294,295]. In addition to neurons and Schwann cells, satellite glial cells, infiltrating immune cells (including infiltrating macrophages), pericytes, endothelial cells, fibro-adipogenic progenitors, adipocytes, fibroblasts, and smooth muscle cells can all be found in skeletal muscle [296,297,298,299].

### 10.2. Overview of Neuromuscular Junction Denervation/Reinnervation

Following a peripheral nerve injury, Wallerian Degeneration occurs, which includes degeneration of the axon terminal and, therefore, denervation from the muscle. Peripheral macrophages and terminal Schwann cells aid in the removal of myelin and neuronal debris. Despite denervation, the post-junctional receptors along the NMJ remain intact, being anchored to basal laminae and covered by terminal Schwann cells [300]. Terminal Schwann cells have been shown to extend processes to neighboring intact NMJs to aid in motor axon sprouting and reinnervation of the denervated synapse, while recruiting immune cells to the NMJ through the release of cytokines [276,301]. With increasing time of denervation, the area of terminal Schwann cell coverage declines, accompanied by a loss of post-synaptic acetylcholine receptors and NMJ fragmentation [300,301,302]. Upon axon regeneration, once the axon has reached its target muscle, terminal Schwann cells aid in guiding the regenerating axon to/through the denervated NMJ [303]. Currently, it is unknown if terminal Schwann cells express TREM2 like the myelinating Schwann cells along peripheral axons. If either infiltrating peripheral macrophages or terminal Schwann cells near the NMJ utilize TREM2 to aid in promoting muscle reinnervation, TREM2 may be used as a therapeutic target for increased functional recovery following peripheral nerve injury, or to aid in conditions such as ALS, where muscle reinnervation does not normally occur.

### 10.3. TREM2 and the Neuromuscular Junction

TREM2+ macrophages have been identified in aged, diseased, and injured skeletal muscle tissue [304,305,306], but to date, they are more often associated with muscle fiber health rather than NMJ mechanisms specifically. RNA sequencing analysis on human muscle tissue identified a subset of lipid-associated macrophages only present in aged tissue that had elevated *TREM2* gene expression and enriched GO terms for phagocytosis and lipid metabolism processes. These cells also had elevated *SPP1* (Osteopontin/Secreted Phosphoprotein 1), which is known to promote fibrosis, suggesting this macrophage subtype may be involved in age-related muscle decline [304]. In a mouse model of volumetric muscle loss with persistent inflammation, spatial transcriptomics identified “Scar-Associated Macrophages” with high *Trem2* and *Spp1* levels as the main driver of persistent inflammation and fibrosis [305]. However, the specific ligands and role of TREM2 in the macrophage’s response to injury were not identified. Recent literature suggests a protective role of TREM2 in skeletal muscle homeostasis, as mutant TREM2 results in altered skeletal muscle composition and strength in mice [307]. Along these lines, Tacconi et al. (2025) published a novel in vitro study illustrating that bone marrow-derived macrophages acquire a lipid-associated macrophage/TREM2+ phenotype following exposure to palmitate and oleate (1:2, “FFA”) [308]. These TREM2+ macrophages consume and store the lipids, suggesting a protective role for their environment, but they also induce transcriptomic changes in neighboring macrophages and skeletal muscles by releasing extracellular vesicles. Extracellular vesicle release subsequently causes increased expression of TREM2 and IL-10 in neighboring macrophages, as well as altered insulin sensitivity, mitochondrial metabolism, and extracellular matrix components in skeletal muscle cells, suggesting tissue remodeling. Collectively, this evidence suggests that TREM2+ macrophages in muscle work to maintain muscle homeostasis but may contribute to fibrosis and muscle alterations with disease or injury.

Despite the current lack of knowledge regarding TREM2 involvement with NMJs specifically, a vast body of literature exists on changes in macrophage activity at the NMJ in injury and disease [276]. The number of macrophages in muscle and their association with the NMJ increased following peripheral nerve injury [276,309] and with ALS disease progression [310,311]. Peripheral nerve injuries performed in mice that lack *Ccr2* have impaired macrophage recruitment to the muscle and diminish muscle function with time, suggesting that infiltrating macrophages are beneficial for muscle reinnervation and functional recovery [309]. In rodent models of SMA, the number of macrophages in muscle dwindles with disease progression. This may indicate a beneficial role for macrophages or simply correlate with the progressive loss of muscle innervation [312]. It is currently unknown if the suggested benefits of infiltrating macrophages into muscle and their association with NMJs in injury or disease involve TREM2 signaling.

## 11. TREM2 as a Therapeutic Target

We hope we conveyed that TREM2 can have beneficial or detrimental effects depending on the cell type and microenvironment, animal model, age of the organism, kind of disease or injury, and stage of disease or injury. This implies that any TREM2 therapeutics require specificity on cell type, spatial, and temporal targeting. As recently reviewed, TREM2 activity can be modulated via (1) enhancing or blocking TREM2 ligands, (2) introducing agonist or antagonist antibodies, (3) targeting ADAM10/17 to promote or diminish sTREM2, (4) modulating gene expression, or (5) targeting downstream targets (e.g., pSYK) [313]. Several agonist or antagonist TREM2 antibodies capable of crossing the BBB are currently in or have previously been in phase one and two clinical trials [28]. Although many of these antibodies have shown success at modulating microglia and provide some degree of temporal specificity due to antibody half-lives, there is limited research suggesting cell-type or spatial specificity, which may result in off-target and potentially detrimental effects over time. Additional studies should be performed on a whole-organismal level and in various disease/injury models to elucidate the efficacy of TREM2 therapeutic antibodies. The utilization of recombinant Adeno-Associated Virus (rAAV) gene therapy to target TREM2 may provide cell type specificity at the cost of temporal controls due to permanent gene editing. New transgenes and viral vectors, including temporal controls using clinically approved doxycycline doses, will need to be developed [314]. Other current work is being performed to improve rAAV tropism for myeloid cells, such as microglia, which have notoriously been difficult to transfect [315]. PR009 (LY3884965) is a current rAAV gene therapy in Phase 1/2a clinical trials for a rare neurodegenerative disorder, adult-onset leukoencephalopathy with axonal spheroids and pigmented glia (ALSP), which is caused by mutations in *Csf1r*. PR009 is suggested to increase *TREM2* expression in microglia and, therefore, may be a potential therapeutic intervention for other neurodegenerative conditions with *TREM2* mutation risk factors [316]. Further, PR009 may be a valuable tool for pre-clinical research to decouple central versus peripheral implications of *TREM2* in conditions such as ALS, spinal cord injury, and peripheral nerve injury. By having cell type, spatial, and temporal specificity of TREM2 targeting, the field may finally unravel the complexity of TREM2’s implication throughout the body in health, aging, disease, and injury.

## 12. Conclusions

Functioning neuronal circuitry between the spinal cord and the periphery is crucial for movement, sensation, autonomic function, and the ability to respond to one’s environment. Aging, injury, and disease all have different implications on spinal reflex circuitry, but all present with neuroinflammation. TREM2 is an immune-modulatory receptor found in myeloid cells in both the CNS and PNS, and more specifically, along all anatomical regions of spinal reflex circuitry. Depending on cell type, microenvironment, age, and type of disease or injury, TREM2 can be beneficial or detrimental (Figure 1, Table 2). Due to the complexities of TREM2 on a whole-organismal level, the development of cell-type, spatial, and temporal specificity for visualizing and modulating TREM2 activity is crucial for pre-clinical research to unravel CNS versus PNS TREM2 effects on health, aging, disease, and injury, and it is imperative for the development of efficient therapeutic advances with minimal off-target effects.

**Table 2:** Summarization of the published effects of TREM2 modulation along spinal cord circuits.

Pathology	Species	Modulation	Effect	Reference
Peripheral Nerve Injury	Mouse	TREM2 knockout	Reduced phagocytosis in DAM-like and female microglia, motoneuron chromatolytic reaction, muscle reinnervation	[142,146]
		DAP_12_ knockout	Reduced allodynia, pro-inflammatory cytokines, motoneuron cell death	[60,186,209]
		Intrathecal TREM2 agonist	Increased allodynia and DAP_12_	[186]
	Rat	Intrathecal TREM2 lentivirus overexpression	Increased allodynia	[187]
Diabetes	Mouse	Intrathecal TREM2 lentivirus overexpression	Increased microglia number, phagocytosis, pro-inflammatory cytokines	[193]
		Intrathecal TREM2 neutralizing antibody	Reduced microglia number, phagocytosis, pro-inflammatory cytokines	[193]
Bone fracture	Mouse	TREM2 Inhibition via Artesunate	Reduced allodynia	[196]
Chemotherapy	Mouse	Intrathecal TREM2 neutralizing antibody	Reduced pro-inflammatory cytokines, allodynia, and peripheral small nerve fiber loss	[199]
Amyotrophic Lateral Sclerosis	Mouse (SOD1 mutant)	TREM2 knockout	Reduced disease-associated microglia, increased homeostatic microglia	[91,146]
	Mouse (AAV- overexpression of hTDP-43)	TREM2 knockout	Increased protein aggregation/reduced TDP-43 clearance, more rapid disease progression, increased loss of motor function	[53]
Spinal Cord Injury	Mouse	TREM2 knockout	Improved microglia autophagy and lysosomal pathways at seven days post injury, reduced microglia phagocytosis, reduced glial scar formation, reduced lesion size, improved locomotion	[79,250]
	BV2 Cells	TREM2 siRNA	Improved microglia autophagy and mitochondrial metabolism	[250]
Health	Primary mouse Schwann Cells	Lentiviral TREM2 knockdown	In Schwann cells: impaired PI3K-AKT-mTOR signaling, activated AMPK and caspases, mitochondrial damage, impaired mitochondrial metabolism, increased apoptosis	[25]
	Mouse	Mutant TREM2	Altered skeletal muscle composition and strength	[307]
Acute Motor Axonal Neuropathy	Mouse	TREM2 knockout	Decreased nerve conduction, myelin debris clearance, axon regeneration, and increased Schwann cell apoptosis	[25]

## Data Availability

This review contains no new data.

## Abbreviations

5XFAD	5 familial Alzheimer’s disease mutations
aa	Amino acids
AD	Alzheimer’s disease
ADAM10	A Disintegrin and Metalloproteinase domain-containing protein 10
ADAM17	A Disintegrin and Metalloproteinase domain-containing protein 17
ADI-R	Autism Diagnostic Interview-Revised
AKT	Protein kinase B
ALS	Amyotrophic Lateral Sclerosis
ALSP	Adult-onset leukoencephalopathy with axonal spheroids and pigmented glia
AMAN	Acute Motor Axonal Neuropathy
AMPK	AMP-activated protein kinase
APOE	Apolipoprotein E
app1	Antiphagocytic Protein 1
arg1	Arginase 1
BBB	Blood–Brain Barrier
C1q	Complement Component 1q
CCL21	CC chemokine ligand 21/6Ckine
ccr2	Chemokine (C-C motif) Receptor 2
CKO	Conditional Knockout
Clec7a	C-type lectin domain family 7, member A.
CNS	Central Nervous System
CSF1	Colony Stimulating Factor 1
CSF1-R	Colony Stimulating Factor 1 Receptor
CTSD	Cathepsin D
CX3CL1	Fractalkine
CX3CR1	C-X3-C motif chemokine receptor 1/Fractalkine receptor
DAM	Disease Associate Microglia
DAP10/Hcst	DNAX activating protein of 10 KD
DAP12/tryobp	DNAX activating protein of 12 KD
DRG	Dorsal Root Ganglion
ePtdSer	Externalized Phosphatidylserine
ERK	Extracellular Signal-Regulated Kinase
GKO	Global Knockout
IL-10	Interleukin-10
IL-1β	Interleukin-1 β
IL-34	Interleukin-34
IL-6	Interleukin-6
ITAM	Immunoreceptor Tyrosine-based Activation Motif.
KV2.1	Potassium Voltage-Gated Channel, Shab-Related Subfamily, Member 1.
LAMP1	Lysosome Associated Membrane Protein 1
LPL	Lipoprotein Lipase
LPS	Lysophosphatidylcholine
MAPK	Mitogen-Activated Protein Kinase
MEK	Mitogen-activated protein kinase kinase
MND	Motor Neuron Disease
mTOR	Mechanistic target of rapamycin/mammalian target of rapamycin
NF-*κ*B	Nuclear Factor kappa-light-chain enhancer of activated B cells
NF2	Merline
NMJ	Neuromuscular Junction
olfml3	Olfactomedin-like 3
P2RX4	P2X purinoceptor 4
P2RY12	Purinergic Receptor P2Y, G Protein Coupled, 12
PD	Parkinson’s disease
PI3K	Phosphatidylinositol 3-kinase.
PLC*γ*	Phospholipase C
PNS	Peripheral Nervous System
PtdSer	Phosphatidylserine
PYK2	Proline-rich tyrosine kinase 2
Raf	Rapidly Accelerated Fibrosarcoma
RAS	Reticular Activating System
RNA	Ribonucleic acid
SHIP1	Src homology 2 (SH2) domain-containing inositol polyphosphate 5-phosphatase 1.
SMA	Spinal Muscular Atrophy
SOD1	Superoxide Dismutase 1.
spp1	Secreted Phosphoprotein 1.
sTREM2	Soluble Triggering Receptor Expressed on Myeloid Cells 2
STZ	Streptozotocin
SYK	Spleen Tyrosine Kinase
TAMS	Tumor-Associated Macrophages
TDP-43	TAR DNA-binding protein 43
TGF-β	Transforming Growth Factor beta
Tmem119	Transmembrane protein 119
TNFα	Tumor Necrosis Factor-alpha
TREM2	Triggering Receptor Expressed on Myeloid Cells 2
VAV2/3	Vav family of guanine nucleotide exchange factors 2/3
VEGFA	Vascular Endothelial Growth Factor A

## References

[1] Philips T., Robberecht W. (2011). Neuroinflammation in amyotrophic lateral sclerosis: Role of glial activation in motor neuron disease. Lancet Neurol..

[2] Liu J., Wang F. (2017). Role of neuroinflammation in amyotrophic lateral sclerosis: Cellular mechanisms and therapeutic implications. Front. Immunol..

[3] Hooten K.G., Beers D.R., Zhao W., Appel S.H. (2015). Protective and toxic neuroinflammation in amyotrophic lateral sclerosis. Neurotherapeutics.

[4] Brennan F.H., Swarts E.A., Kigerl K.A., Mifflin K.A., Guan Z., Noble B.T., Wang Y., Witcher K.G., Godbout J.P., Popovich P.G. (2024). Microglia promote maladaptive plasticity in autonomic circuitry after spinal cord injury in mice. Sci. Transl. Med..

[5] Thompson A.K., Sinkjær T., Rajendram R., Preedy V.R., Martin C.R. (2022). Chapter 30—Features and physiology of spinal stretch reflexes in people with chronic spinal cord injury. Cellular, Molecular, Physiological, and Behavioral Aspects of Spinal Cord Injury.

[6] Li C., Xiong W., Wan B., Kong G., Wang S., Wang Y., Fan J. (2022). Role of peripheral immune cells in spinal cord injury. Cell. Mol. Life Sci..

[7] DiSabato D.J., Marion C.M., Mifflin K.A., Alfredo A.N., Rodgers K.A., Kigerl K.A., Popovich P.G., McTigue D.M. (2024). System failure: Systemic inflammation following spinal cord injury. Eur. J. Immunol..

[8] Schwab J.M., Zhang Y., Kopp M.A., Brommer B., Popovich P.G. (2014). The paradox of chronic neuroinflammation, systemic immune suppression, autoimmunity after traumatic chronic spinal cord injury. Exp. Neurol..

[9] Pottorf T.S., Rotterman T.M., McCallum W.M., Haley-Johnson Z.A., Alvarez F.J. (2022). The Role of Microglia in Neuroinflammation of the Spinal Cord after Peripheral Nerve Injury. Cells.

[10] Konishi H., Kiyama H. (2018). Microglial TREM2/DAP12 Signaling: A Double-Edged Sword in Neural Diseases. Front. Cell. Neurosci..

[11] Colonna M. (2023). The biology of TREM receptors. Nat. Rev. Immunol..

[12] Colonna M. (2003). TREMs in the immune system and beyond. Nat. Rev. Immunol..

[13] Yang J., Fu Z., Zhang X., Xiong M., Meng L., Zhang Z. (2020). TREM2 ectodomain and its soluble form in Alzheimer’s disease. J. Neuroinflamm..

[14] Lanier L.L. (2009). DAP10-and DAP12-associated receptors in innate immunity. Immunol. Rev..

[15] Allcock R.J., Barrow A.D., Forbes S., Beck S., Trowsdale J. (2003). The human TREM gene cluster at 6p21. 1 encodes both activating and inhibitory single IgV domain receptors and includes NKp44. Eur. J. Immunol..

[16] Kasamatsu J., Deng M., Azuma M., Funami K., Shime H., Oshiumi H., Matsumoto M., Kasahara M., Seya T. (2016). Double-stranded RNA analog and type I interferon regulate expression of Trem paired receptors in murine myeloid cells. BMC Immunol..

[17] Stet R.J., Hermsen T., Westphal A.H., Jukes J., Engelsma M., Lidy Verburg-van Kemenade B., Dortmans J., Aveiro J., Savelkoul H.F. (2005). Novel immunoglobulin-like transcripts in teleost fish encode polymorphic receptors with cytoplasmic ITAM or ITIM and a new structural Ig domain similar to the natural cytotoxicity receptor NKp44. Immunogenetics.

[18] Viertlboeck B.C., Schmitt R., Göbel T.W. (2006). The chicken immunoregulatory receptor families SIRP, TREM, and CMRF35/CD300L. Immunogenetics.

[19] Jin S.C., Benitez B.A., Karch C.M., Cooper B., Skorupa T., Carrell D., Norton J.B., Hsu S., Harari O., Cai Y. (2014). Coding variants in TREM2 increase risk for Alzheimer’s disease. Hum. Mol. Genet..

[20] Jay T.R., von Saucken V.E., Landreth G.E. (2017). TREM2 in neurodegenerative diseases. Mol. Neurodegener..

[21] Shaw B.C., Snider H.C., Turner A.K., Zajac D.J., Simpson J.F., Estus S., Combs C. (2022). An Alternatively Spliced TREM2 Isoform Lacking the Ligand Binding Domain is Expressed in Human Brain. J. Alzheimer’s Dis..

[22] Peng Q., Malhotra S., Torchia J.A., Kerr W.G., Coggeshall K.M., Humphrey M.B. (2010). TREM2-and DAP12-dependent activation of PI3K requires DAP10 and is inhibited by SHIP1. Sci. Signal..

[23] Wunderlich P., Glebov K., Kemmerling N., Tien N.T., Neumann H., Walter J. (2013). Sequential proteolytic processing of the triggering receptor expressed on myeloid cells-2 (TREM2) protein by ectodomain shedding and γ-secretase-dependent intramembranous cleavage. J. Biol. Chem..

[24] Lin M., Yu J.X., Zhang W.X., Lao F.X., Huang H.C. (2024). Roles of TREM2 in the Pathological Mechanism and the Therapeutic Strategies of Alzheimer’s Disease. J. Prev. Alzheimer’s Dis..

[25] Zhang N., Ji Q., Chen Y., Wen X., Shan F. (2024). TREM2 deficiency impairs the energy metabolism of Schwann cells and exacerbates peripheral neurological deficits. Cell Death Dis..

[26] Sun H., Feng J., Tang L. (2020). Function of TREM1 and TREM2 in liver-related diseases. Cells.

[27] Lee J.W., Lee I.H., Iimura T., Kong S.W. (2021). Two macrophages, osteoclasts and microglia: From development to pleiotropy. Bone Res..

[28] Wang X., Wang Y., Yang L., Zhang Y., Yang L. (2025). TREM2+ macrophages: A key role in disease development. Front. Immunol..

[29] Ulland T.K., Colonna M. (2018). TREM2—A key player in microglial biology and Alzheimer disease. Nat. Rev. Neurol..

[30] Colonna M., Wang Y. (2016). TREM2 variants: New keys to decipher Alzheimer disease pathogenesis. Nat. Rev. Neurosci..

[31] Hansen D.V., Hanson J.E., Sheng M. (2018). Microglia in Alzheimer’s disease. J. Cell Biol..

[32] Painter M.M., Atagi Y., Liu C.C., Rademakers R., Xu H., Fryer J.D., Bu G. (2015). TREM2 in CNS homeostasis and neurodegenerative disease. Mol. Neurodegener..

[33] Tang D., Kang R., Coyne C.B., Zeh H.J., Lotze M.T. (2012). PAMPs and DAMPs: Signal 0s that spur autophagy and immunity. Immunol. Rev..

[34] Brantley-Sieders D.M., Zhuang G., Vaught D., Freeman T., Hwang Y., Hicks D., Chen J. (2009). Host deficiency in Vav2/3 guanine nucleotide exchange factors impairs tumor growth, survival, and angiogenesis in vivo. Mol. Cancer Res..

[35] Li C., Götz J. (2018). Pyk2 is a Novel Tau Tyrosine Kinase that is Regulated by the Tyrosine Kinase Fyn. J. Alzheimer’s Dis..

[36] Martelli M.P., Lin H., Zhang W., Samelson L.E., Bierer B.E. (2000). Signaling via LAT (linker for T-cell activation) and Syk/ZAP70 is required for ERK activation and NFAT transcriptional activation following CD2 stimulation. Blood.

[37] Tan M.S., Yu J.T., Tan L. (2013). Bridging integrator 1 (BIN1): Form, function, and Alzheimer’s disease. Trends Mol. Med..

[38] Griciuc A., Patel S., Federico A.N., Choi S.H., Innes B.J., Oram M.K., Cereghetti G., McGinty D., Anselmo A., Sadreyev R.I. (2019). TREM2 Acts Downstream of CD33 in Modulating Microglial Pathology in Alzheimer’s Disease. Neuron.

[39] Cheng B., Li X., Dai K., Duan S., Rong Z., Chen Y., Lü L., Liu Z., Huang X., Xu H. (2021). Triggering receptor expressed on myeloid cells-2 (TREM2) interacts with colony-stimulating factor 1 receptor (CSF1R) but is not necessary for CSF1/CSF1R-mediated microglial survival. Front. Immunol..

[40] Zhang L., Xiang X., Li Y., Bu G., Chen X.F. (2025). TREM2 and sTREM2 in Alzheimer’s disease: From mechanisms to therapies. Mol. Neurodegener..

[41] Mócsai A., Ruland J., Tybulewicz V.L. (2010). The SYK tyrosine kinase: A crucial player in diverse biological functions. Nat. Rev. Immunol..

[42] Wang Y., Cella M., Mallinson K., Ulrich J.D., Young K.L., Robinette M.L., Gilfillan S., Krishnan G.M., Sudhakar S., Zinselmeyer B.H. (2015). TREM2 lipid sensing sustains the microglial response in an Alzheimer’s disease model. Cell.

[43] Shirotani K., Hori Y., Yoshizaki R., Higuchi E., Colonna M., Saito T., Hashimoto S., Saito T., Saido T.C., Iwata N. (2019). Aminophospholipids are signal-transducing TREM2 ligands on apoptotic cells. Sci. Rep..

[44] Kober D.L., Stuchell-Brereton M.D., Kluender C.E., Dean H.B., Strickland M.R., Steinberg D.F., Nelson S.S., Baban B., Holtzman D.M., Frieden C. (2021). Functional insights from biophysical study of TREM2 interactions with apoE and Aβ1-42. Alzheimer’s Dement..

[45] Cannon J.P., O’Driscoll M., Litman G.W. (2012). Specific lipid recognition is a general feature of CD300 and TREM molecules. Immunogenetics.

[46] Song W., Hooli B., Mullin K., Jin S.C., Cella M., Ulland T.K., Wang Y., Tanzi R.E., Colonna M. (2017). Alzheimer’s disease-associated TREM2 variants exhibit either decreased or increased ligand-dependent activation. Alzheimer’s Dement..

[47] Atagi Y., Liu C.C., Painter M.M., Chen X.F., Verbeeck C., Zheng H., Li X., Rademakers R., Kang S.S., Xu H. (2015). Apolipoprotein E Is a Ligand for Triggering Receptor Expressed on Myeloid Cells 2 (TREM2). J. Biol. Chem..

[48] Bailey C.C., DeVaux L.B., Farzan M. (2015). The Triggering Receptor Expressed on Myeloid Cells 2 Binds Apolipoprotein E. J. Biol. Chem..

[49] Kawabori M., Kacimi R., Kauppinen T., Calosing C., Kim J.Y., Hsieh C.L., Nakamura M.C., Yenari M.A. (2015). Triggering receptor expressed on myeloid cells 2 (TREM2) deficiency attenuates phagocytic activities of microglia and exacerbates ischemic damage in experimental stroke. J. Neurosci..

[50] Daws M.R., Sullam P.M., Niemi E.C., Chen T.T., Tchao N.K., Seaman W.E. (2003). Pattern recognition by TREM-2: Binding of anionic ligands. J. Immunol..

[51] Iizasa E.i., Chuma Y., Uematsu T., Kubota M., Kawaguchi H., Umemura M., Toyonaga K., Kiyohara H., Yano I., Colonna M. (2021). TREM2 is a receptor for non-glycosylated mycolic acids of mycobacteria that limits anti-mycobacterial macrophage activation. Nat. Commun..

[52] Zhao Y., Wu X., Li X., Jiang L.L., Gui X., Liu Y., Sun Y., Zhu B., Piña-Crespo J.C., Zhang M. (2018). TREM2 is a receptor for β-amyloid that mediates microglial function. Neuron.

[53] Xie M., Liu Y.U., Zhao S., Zhang L., Bosco D.B., Pang Y.P., Zhong J., Sheth U., Martens Y.A., Zhao N. (2022). TREM2 interacts with TDP-43 and mediates microglial neuroprotection against TDP-43-related neurodegeneration. Nat. Neurosci..

[54] Mills III W.A., Eyo U.B. (2023). TREMble before TREM2: The mighty microglial receptor conferring neuroprotective properties in TDP-43 mediated neurodegeneration. Neurosci. Bull..

[55] Mason L.H., Willette-Brown J., Taylor L.S., McVicar D.W. (2006). Regulation of Ly49D/DAP12 Signal Transduction by Src-Family Kinases and CD4512. J. Immunol..

[56] Alonso G., Koegl M., Mazurenko N., Courtneidge S.A. (1995). Sequence Requirements for Binding of Src Family Tyrosine Kinases to Activated Growth Factor Receptors. J. Biol. Chem..

[57] Luo J., Elwood F., Britschgi M., Villeda S., Zhang H., Ding Z., Zhu L., Alabsi H., Getachew R., Narasimhan R. (2013). Colony-stimulating factor 1 receptor (CSF1R) signaling in injured neurons facilitates protection and survival. J. Exp. Med..

[58] Hamilton J.A. (1997). CSF-1 signal transduction. J. Leukoc. Biol..

[59] Pixley F.J., Stanley E.R. (2004). CSF-1 regulation of the wandering macrophage: Complexity in action. Trends Cell Biol..

[60] Guan Z., Kuhn J.A., Wang X., Colquitt B., Solorzano C., Vaman S., Guan A.K., Evans-Reinsch Z., Braz J., Devor M. (2016). Injured sensory neuron–derived CSF1 induces microglial proliferation and DAP12-dependent pain. Nat. Neurosci..

[61] Otero K., Turnbull I.R., Poliani P.L., Vermi W., Cerutti E., Aoshi T., Tassi I., Takai T., Stanley S.L., Miller M. (2009). Macrophage colony-stimulating factor induces the proliferation and survival of macrophages via a pathway involving DAP12 and β-catenin. Nat. Immunol..

[62] Thornton P., Sevalle J., Deery M.J., Fraser G., Zhou Y., Ståhl S., Franssen E.H., Dodd R.B., Qamar S., Gomez Perez-Nievas B. (2017). TREM2 shedding by cleavage at the H157-S158 bond is accelerated for the Alzheimer’s disease-associated H157Y variant. EMBO Mol. Med..

[63] Schlepckow K., Kleinberger G., Fukumori A., Feederle R., Lichtenthaler S.F., Steiner H., Haass C. (2017). An Alzheimer-associated TREM2 variant occurs at the ADAM cleavage site and affects shedding and phagocytic function. EMBO Mol. Med..

[64] Glebov K., Wunderlich P., Karaca I., Walter J. (2016). Functional involvement of γ-secretase in signaling of the triggering receptor expressed on myeloid cells-2 (TREM2). J. Neuroinflamm..

[65] Feuerbach D., Schindler P., Barske C., Joller S., Beng-Louka E., Worringer K.A., Kommineni S., Kaykas A., Ho D.J., Ye C. (2017). ADAM17 is the main sheddase for the generation of human triggering receptor expressed in myeloid cells (hTREM2) ectodomain and cleaves TREM2 after Histidine 157. Neurosci. Lett..

[66] Filipello F., Goldsbury C., You S.F., Locca A., Karch C.M., Piccio L. (2022). Soluble TREM2: Innocent bystander or active player in neurological diseases?. Neurobiol. Dis..

[67] Moutinho M., Coronel I., Tsai A.P., Di Prisco G.V., Pennington T., Atwood B.K., Puntambekar S.S., Smith D.C., Martinez P., Han S. (2023). TREM2 splice isoforms generate soluble TREM2 species that disrupt long-term potentiation. Genome Med..

[68] Moutinho M., Landreth G.E. (2021). TREM2 splicing emerges as crucial aspect to understand TREM2 biology. J. Leukoc. Biol..

[69] Piccio L., Buonsanti C., Cella M., Tassi I., Schmidt R.E., Fenoglio C., Rinker J., Naismith R.T., Panina-Bordignon P., Passini N. (2008). Identification of soluble TREM-2 in the cerebrospinal fluid and its association with multiple sclerosis and CNS inflammation. Brain J. Neurol..

[70] Jericó I., Vicuña-Urriza J., Blanco-Luquin I., Macias M., Martinez-Merino L., Roldán M., Rojas-Garcia R., Pagola-Lorz I., Carbayo A., De Luna N. (2023). Profiling TREM2 expression in amyotrophic lateral sclerosis. Brain Behav. Immun..

[71] Zhong L., Chen X.F., Wang T., Wang Z., Liao C., Wang Z., Huang R., Wang D., Li X., Wu L. (2017). Soluble TREM2 induces inflammatory responses and enhances microglial survival. J. Exp. Med..

[72] Song W.M., Joshita S., Zhou Y., Ulland T.K., Gilfillan S., Colonna M. (2018). Humanized TREM2 mice reveal microglia-intrinsic and -extrinsic effects of R47H polymorphism. J. Exp. Med..

[73] Wu K., Byers D.E., Jin X., Agapov E., Alexander-Brett J., Patel A.C., Cella M., Gilfilan S., Colonna M., Kober D.L. (2015). TREM-2 promotes macrophage survival and lung disease after respiratory viral infection. J. Exp. Med..

[74] Park S.H., Lee E.H., Kim H.J., Jo S., Lee S., Seo S.W., Park H.H., Koh S.H., Lee J.H. (2021). The relationship of soluble TREM2 to other biomarkers of sporadic Alzheimer’s disease. Sci. Rep..

[75] Nabizadeh F., Seyedmirzaei H., Karami S. (2024). Neuroimaging biomarkers and CSF sTREM2 levels in Alzheimer’s disease: A longitudinal study. Sci. Rep..

[76] Wang R., Zhan Y., Zhu W., Yang Q., Pei J. (2024). Association of soluble TREM2 with Alzheimer’s disease and mild cognitive impairment: A systematic review and meta-analysis. Front. Aging Neurosci..

[77] Jiao L., Yang J., Wang W., Liu X., Fu Y., Fan D. (2024). sTREM2 cerebrospinal fluid levels are a potential biomarker in amyotrophic lateral sclerosis and associate with UMN burden. Front. Neurol..

[78] Cooper-Knock J., Green C., Altschuler G., Wei W., Bury J.J., Heath P.R., Wyles M., Gelsthorpe C., Highley J.R., Lorente-Pons A. (2017). A data-driven approach links microglia to pathology and prognosis in amyotrophic lateral sclerosis. Acta Neuropathol. Commun..

[79] Gao H., Di J., Clausen B.H., Wang N., Zhu X., Zhao T., Chang Y., Pang M., Yang Y., He R. (2023). Distinct myeloid population phenotypes dependent on TREM2 expression levels shape the pathology of traumatic versus demyelinating CNS disorders. Cell Rep..

[80] Paloneva J., Manninen T., Christman G., Hovanes K., Mandelin J., Adolfsson R., Bianchin M., Bird T., Miranda R., Salmaggi A. (2002). Mutations in Two Genes Encoding Different Subunits of a Receptor Signaling Complex Result in an Identical Disease Phenotype. Am. J. Hum. Genet..

[81] Paloneva J., Mandelin J., Kiialainen A., Böhling T., Prudlo J., Hakola P., Haltia M., Konttinen Y.T., Peltonen L. (2003). DAP12/TREM2 deficiency results in impaired osteoclast differentiation and osteoporotic features. J. Exp. Med..

[82] Zhou Y., Tada M., Cai Z., Andhey P.S., Swain A., Miller K.R., Gilfillan S., Artyomov M.N., Takao M., Kakita A. (2023). Human early-onset dementia caused by DAP12 deficiency reveals a unique signature of dysregulated microglia. Nat. Immunol..

[83] Kober D.L., Alexander-Brett J.M., Karch C.M., Cruchaga C., Colonna M., Holtzman M.J., Brett T.J. (2016). Neurodegenerative disease mutations in TREM2 reveal a functional surface and distinct loss-of-function mechanisms. eLife.

[84] Guerreiro R., Wojtas A., Bras J., Carrasquillo M., Rogaeva E., Majounie E., Cruchaga C., Sassi C., Kauwe J.S., Younkin S. (2013). TREM2 variants in Alzheimer’s disease. N. Engl. J. Med..

[85] Borroni B., Ferrari F., Galimberti D., Nacmias B., Barone C., Bagnoli S., Fenoglio C., Piaceri I., Archetti S., Bonvicini C. (2014). Heterozygous TREM2 mutations in frontotemporal dementia. Neurobiol. Aging.

[86] Ogonowski N., Santamaria-Garcia H., Baez S., Lopez A., Laserna A., Garcia-Cifuentes E., Ayala-Ramirez P., Zarante I., Suarez-Obando F., Reyes P. (2023). Frontotemporal dementia presentation in patients with heterozygous p. H157Y variant of TREM2. J. Med. Genet..

[87] Jonsson T., Stefansson H., Steinberg S., Jonsdottir I., Jonsson P.V., Snaedal J., Bjornsson S., Huttenlocher J., Levey A.I., Lah J.J. (2013). Variant of TREM2 associated with the risk of Alzheimer’s disease. N. Engl. J. Med..

[88] Zhu B., Liu Y., Hwang S., Archuleta K., Huang H., Campos A., Murad R., Piña-Crespo J., Xu H., Huang T.Y. (2022). Trem2 deletion enhances tau dispersion and pathology through microglia exosomes. Mol. Neurodegener..

[89] Popescu A.S., Butler C.A., Allendorf D.H., Piers T.M., Mallach A., Roewe J., Reinhardt P., Cinti A., Redaelli L., Boudesco C. (2023). Alzheimer’s disease-associated R47H TREM2 increases, but wild-type TREM2 decreases, microglial phagocytosis of synaptosomes and neuronal loss. Glia.

[90] Zhou S.L., Tan C.C., Hou X.H., Cao X.P., Tan L., Yu J.T. (2019). TREM2 Variants and Neurodegenerative Diseases: A Systematic Review and Meta-Analysis. J. Alzheimer’s Dis..

[91] Krasemann S., Madore C., Cialic R., Baufeld C., Calcagno N., El Fatimy R., Beckers L., O’Loughlin E., Xu Y., Fanek Z. (2017). The TREM2-APOE Pathway Drives the Transcriptional Phenotype of Dysfunctional Microglia in Neurodegenerative Diseases. Immunity.

[92] Gratuze M., Leyns C.E.G., Holtzman D.M. (2018). New insights into the role of TREM2 in Alzheimer’s disease. Mol. Neurodegener..

[93] Rayaprolu S., Mullen B., Baker M., Lynch T., Finger E., Seeley W.W., Hatanpaa K.J., Lomen-Hoerth C., Kertesz A., Bigio E.H. (2013). TREM2 in neurodegeneration: Evidence for association of the p. R47H variant with frontotemporal dementia and Parkinson’s disease. Mol. Neurodegener..

[94] Cady J., Koval E.D., Benitez B.A., Zaidman C., Jockel-Balsarotti J., Allred P., Baloh R.H., Ravits J., Simpson E., Appel S.H. (2014). TREM2 variant p. R47H as a risk factor for sporadic amyotrophic lateral sclerosis. JAMA Neurol..

[95] Peplonska B., Berdynski M., Mandecka M., Barczak A., Kuzma-Kozakiewicz M., Barcikowska M., Zekanowski C. (2018). TREM2 variants in neurodegenerative disorders in the Polish population. Homozygosity and compound heterozygosity in FTD patients. Amyotroph. Lateral Scler. Front. Degener..

[96] Lu Y., Liu W., Wang X. (2015). TREM2 variants and risk of Alzheimer’s disease: A meta-analysis. Neurol. Sci..

[97] Guerreiro R.J., Lohmann E., Brás J.M., Gibbs J.R., Rohrer J.D., Gurunlian N., Dursun B., Bilgic B., Hanagasi H., Gurvit H. (2013). Using exome sequencing to reveal mutations in TREM2 presenting as a frontotemporal dementia–like syndrome without bone involvement. JAMA Neurol..

[98] Bock V., Botturi A., Gaviani P., Lamperti E., Maccagnano C., Piccio L., Silvani A., Salmaggi A. (2013). Polycystic lipomembranous osteodysplasia with sclerosing leukoencephalopathy (PLOSL): A new report of an Italian woman and review of the literature. J. Neurol. Sci..

[99] Klunemann H., Ridha B., Magy L., Wherrett J., Hemelsoet D., Keen R., De Bleecker J., Rossor M., Marienhagen J., Klein H. (2005). The genetic causes of basal ganglia calcification, dementia, and bone cysts: DAP12 and TREM2. Neurology.

[100] Soragna D., Tupler R., Ratti M., Montalbetti L., Papi L., Sestini R. (2003). An Italian family affected by Nasu-Hakola disease with a novel genetic mutation in the TREM2 gene. J. Neurol. Neurosurg. Psychiatry.

[101] Samanci B., Bilgiç B., Gelişin Ö., Tepgeç F., Guven G., Tüfekçioğlu Z., Alaylıoğlu M., Hanagasi H.A., Gürvit H., Guerreiro R. (2021). TREM2 variants as a possible cause of frontotemporal dementia with distinct neuroimaging features. Eur. J. Neurol..

[102] Numasawa Y., Yamaura C., Ishihara S., Shintani S., Yamazaki M., Tabunoki H., Satoh J.I. (2011). Nasu–Hakola disease with a splicing mutation of TREM2 in a Japanese family. Eur. J. Neurol..

[103] Dijkstra J.I.R., Vermunt L., Venkatraghavan V., Ozhegov G., Coomans E.M., Ossenkoppele R., van de Giessen E., Hulsman M., de Geus C.M., van der Flier W.M. (2025). TREM2 risk variants and associated endophenotypes in alzheimer’s disease. Alzheimer’s Res. Ther..

[104] Dardiotis E., Siokas V., Pantazi E., Dardioti M., Rikos D., Xiromerisiou G., Markou A., Papadimitriou D., Speletas M., Hadjigeorgiou G.M. (2017). A novel mutation in TREM2 gene causing Nasu-Hakola disease and review of the literature. Neurobiol. Aging.

[105] Roussos P., Katsel P., Fam P., Tan W., Purohit D.P., Haroutunian V. (2015). The triggering receptor expressed on myeloid cells 2 (TREM2) is associated with enhanced inflammation, neuropathological lesions and increased risk for Alzheimer’s dementia. Alzheimer’s Dement..

[106] Le Ber I., De Septenville A., Guerreiro R., Bras J., Camuzat A., Caroppo P., Lattante S., Couarch P., Kabashi E., Bouya-Ahmed K. (2014). Homozygous TREM2 mutation in a family with atypical frontotemporal dementia. Neurobiol. Aging.

[107] Giraldo M., Lopera F., Siniard A.L., Corneveaux J.J., Schrauwen I., Carvajal J., Muñoz C., Ramirez-Restrepo M., Gaiteri C., Myers A.J. (2013). Variants in triggering receptor expressed on myeloid cells 2 are associated with both behavioral variant frontotemporal lobar degeneration and Alzheimer’s disease. Neurobiol. Aging.

[108] Guerreiro R., Bilgic B., Guven G., Brás J., Rohrer J., Lohmann E., Hanagasi H., Gurvit H., Emre M. (2013). A novel compound heterozygous mutation in TREM2 found in a Turkish frontotemporal dementia-like family. Neurobiol. Aging.

[109] Thelen M., Razquin C., Hernández I., Gorostidi A., Sanchez-Valle R., Ortega-Cubero S., Wolfsgruber S., Drichel D., Fliessbach K., Duenkel T. (2014). Investigation of the role of rare TREM2 variants in frontotemporal dementia subtypes. Neurobiol. Aging.

[110] Jiang T., Tan L., Chen Q., Tan M.S., Zhou J.S., Zhu X.C., Lu H., Wang H.F., Zhang Y.D., Yu J.T. (2016). A rare coding variant in TREM2 increases risk for Alzheimer’s disease in Han Chinese. Neurobiol. Aging.

[111] Jin S.C., Carrasquillo M.M., Benitez B.A., Skorupa T., Carrell D., Patel D., Lincoln S., Krishnan S., Kachadoorian M., Reitz C. (2015). TREM2 is associated with increased risk for Alzheimer’s disease in African Americans. Mol. Neurodegener..

[112] Kang S.S., Kurti A., Baker K.E., Liu C.C., Colonna M., Ulrich J.D., Holtzman D.M., Bu G., Fryer J.D. (2018). Behavioral and transcriptomic analysis of Trem2-null mice: Not all knockout mice are created equal. Hum. Mol. Genet..

[113] Filipello F., Morini R., Corradini I., Zerbi V., Canzi A., Michalski B., Erreni M., Markicevic M., Starvaggi-Cucuzza C., Otero K. (2018). The microglial innate immune receptor TREM2 is required for synapse elimination and normal brain connectivity. Immunity.

[114] Tagliatti E., Desiato G., Mancinelli S., Bizzotto M., Gagliani M.C., Faggiani E., Hernández-Soto R., Cugurra A., Poliseno P., Miotto M. (2024). Trem2 expression in microglia is required to maintain normal neuronal bioenergetics during development. Immunity.

[115] Schmid C.D., Sautkulis L.N., Danielson P.E., Cooper J., Hasel K.W., Hilbush B.S., Sutcliffe J.G., Carson M.J. (2002). Heterogeneous expression of the triggering receptor expressed on myeloid cells-2 on adult murine microglia. J. Neurochem..

[116] Forabosco P., Ramasamy A., Trabzuni D., Walker R., Smith C., Bras J., Levine A.P., Hardy J., Pocock J.M., Guerreiro R. (2013). Insights into TREM2 biology by network analysis of human brain gene expression data. Neurobiol. Aging.

[117] Leyns C.E., Ulrich J.D., Finn M.B., Stewart F.R., Koscal L.J., Remolina Serrano J., Robinson G.O., Anderson E., Colonna M., Holtzman D.M. (2017). TREM2 deficiency attenuates neuroinflammation and protects against neurodegeneration in a mouse model of tauopathy. Proc. Natl. Acad. Sci. USA.

[118] Leyns C.E., Gratuze M., Narasimhan S., Jain N., Koscal L.J., Jiang H., Manis M., Colonna M., Lee V.M., Ulrich J.D. (2019). TREM2 function impedes tau seeding in neuritic plaques. Nat. Neurosci..

[119] Skarnes W.C., Rosen B., West A.P., Koutsourakis M., Bushell W., Iyer V., Mujica A.O., Thomas M., Harrow J., Cox T. (2011). A conditional knockout resource for the genome-wide study of mouse gene function. Nature.

[120] Brierley J.B. (1950). The penetration of particulate matter from the cerebrospinal fluid into the spinal ganglia, peripheral nerves, and perivascular spaces of the central nervous system. J. Neurol. Neurosurg. Psychiatry.

[121] Devor M. (1999). Unexplained peculiarities of the dorsal root ganglion. PAIN.

[122] Nagashima K., Oota K. (1974). A Histopathological Study of the Human Spinal Ganglia. Normal Variations in Aging. Acta Patholigica Jpn..

[123] Tandrup T. (2004). Unbiased estimates of number and size of rat dorsal root ganglion cells in studies of structure and cell survival. J. Neurocytol..

[124] West C.A., McKay Hart A., Terenghi G., Wiberg M. (2012). Sensory neurons of the human brachial plexus: A quantitative study employing optical fractionation and in vivo volumetric magnetic resonance imaging. Neurosurgery.

[125] Haberberger R.V., Kuramatilake J., Barry C.M., Matusica D. (2023). Ultrastructure of dorsal root ganglia. Cell Tissue Res..

[126] Avraham O., Feng R., Ewan E.E., Rustenhoven J., Zhao G., Cavalli V. (2021). Profiling sensory neuron microenvironment after peripheral and central axon injury reveals key pathways for neural repair. eLife.

[127] Feringa E.R., Lee G.W., Vahlsing H.L., Gilbertie W.J. (1985). Cell death in the adult rat dorsal root ganglion after hind limb amputation, spinal cord transection, or both operations. Exp. Neurol..

[128] Lu S. (2023). Single-Cell Transcriptome Analyses of Dorsal Root Ganglia in Aged Hyperlipidemic Mice. Ph.D. Thesis.

[129] Li X., Zhuang Z., Hao Y., Lin S., Gu J., Chang S., Lan L., Zhao G., Zhang D. (2025). Paeonol Relieves Chronic Neuropathic Pain by Reducing Communication Between Schwann Cells and Macrophages in the Dorsal Root Ganglia After Injury. Int. J. Mol. Sci..

[130] Qu Y., Cai R., Li Q., Wang H., Lu L. (2024). Neuroinflammation signatures in dorsal root ganglia following chronic constriction injury. Heliyon.

[131] Yu X., Liu H., Hamel K.A., Morvan M.G., Yu S., Leff J., Guan Z., Braz J.M., Basbaum A.I. (2020). Dorsal root ganglion macrophages contribute to both the initiation and persistence of neuropathic pain. Nat. Commun..

[132] Feng R., Muraleedharan Saraswathy V., Mokalled M.H., Cavalli V. (2023). Self-renewing macrophages in dorsal root ganglia contribute to promote nerve regeneration. Proc. Natl. Acad. Sci. USA.

[133] Brown A.G. (2012). Organization in the Spinal Cord: The Anatomy and Physiology of Identified Neurones.

[134] Fowler C.J., Griffiths D., De Groat W.C. (2008). The neural control of micturition. Nat. Rev. Neurosci..

[135] Kuru M. (1965). Nervous control of micturition. Physiol. Rev..

[136] Uher E.-M., Swash M. (1998). Sacral reflexes: Physiology and clinical application. Dis. Colon Rectum.

[137] Diaz E., Morales H. (2016). Spinal Cord Anatomy and Clinical Syndromes. Semin. Ultrasound CT MRI.

[138] Cramer G.D., Darby S.A. (2013). Clinical Anatomy of the Spine, Spinal Cord, and ANS.

[139] Green T.R., Rowe R.K. (2024). Quantifying microglial morphology: An insight into function. Clin. Exp. Immunol..

[140] Vidal-Itriago A., Radford R.A., Aramideh J.A., Maurel C., Scherer N.M., Don E.K., Lee A., Chung R.S., Graeber M.B., Morsch M. (2022). Microglia morphophysiological diversity and its implications for the CNS. Front. Immunol..

[141] Edler M.K., Mhatre-Winters I., Richardson J.R. (2021). Microglia in Aging and Alzheimer’s Disease: A Comparative Species Review. Cells.

[142] Pottorf T.S., Lane E.L., Haley-Johnson Z., Amores-Sanchez V., Ukmar D.N., Correa-Torres P.M., Alvarez F.J. (2025). Dual Role of Microglial TREM2 in Neuronal Degeneration and Regeneration After Axotomy. bioRxiv.

[143] Hakim R., Zachariadis V., Sankavaram S.R., Han J., Harris R.A., Brundin L., Enge M., Svensson M. (2021). Spinal Cord Injury Induces Permanent Reprogramming of Microglia into a Disease-Associated State Which Contributes to Functional Recovery. J. Neurosci..

[144] Keren-Shaul H., Spinrad A., Weiner A., Matcovitch-Natan O., Dvir-Szternfeld R., Ulland T.K., David E., Baruch K., Lara-Astaiso D., Toth B. (2017). A unique microglia type associated with restricting development of Alzheimer’s disease. Cell.

[145] Samant R.R., Standaert D.G., Harms A.S. (2024). The emerging role of disease-associated microglia in Parkinson’s disease. Front. Cell. Neurosci..

[146] Xie M., Zhao S., Bosco D.B., Nguyen A., Wu L.J. (2022). Microglial TREM2 in amyotrophic lateral sclerosis. Dev. Neurobiol..

[147] Wang H. (2021). Microglia heterogeneity in Alzheimer’s disease: Insights from single-cell technologies. Front. Synaptic Neurosci..

[148] Deczkowska A., Keren-Shaul H., Weiner A., Colonna M., Schwartz M., Amit I. (2018). Disease-associated microglia: A universal immune sensor of neurodegeneration. Cell.

[149] Zhou Y., Song W.M., Andhey P.S., Swain A., Levy T., Miller K.R., Poliani P.L., Cominelli M., Grover S., Gilfillan S. (2020). Human and mouse single-nucleus transcriptomics reveal TREM2-dependent and TREM2-independent cellular responses in Alzheimer’s disease. Nat. Med..

[150] Lambert J.C., Ibrahim-Verbaas C.A., Harold D., Naj A.C., Sims R., Bellenguez C., Jun G., DeStefano A.L., Bis J.C., Beecham G.W. (2013). Meta-analysis of 74,046 individuals identifies 11 new susceptibility loci for Alzheimer’s disease. Nat. Genet..

[151] Song W.M., Colonna M. (2018). The identity and function of microglia in neurodegeneration. Nat. Immunol..

[152] McQuade A., Kang Y.J., Hasselmann J., Jairaman A., Sotelo A., Coburn M., Shabestari S.K., Chadarevian J.P., Fote G., Tu C.H. (2020). Gene expression and functional deficits underlie TREM2-knockout microglia responses in human models of Alzheimer’s disease. Nat. Commun..

[153] Ennerfelt H., Frost E.L., Shapiro D.A., Holliday C., Zengeler K.E., Voithofer G., Bolte A.C., Lammert C.R., Kulas J.A., Ulland T.K. (2022). SYK coordinates neuroprotective microglial responses in neurodegenerative disease. Cell.

[154] Matteoli M. (2024). The role of microglial TREM2 in development: A path toward neurodegeneration?. Glia.

[155] Jay T.R., von Saucken V.E., Muñoz B., Codocedo J.F., Atwood B.K., Lamb B.T., Landreth G.E. (2019). TREM2 is required for microglial instruction of astrocytic synaptic engulfment in neurodevelopment. Glia.

[156] Vecchiarelli H.A., Bisht K., Sharma K., Weiser Novak S., Traetta M.E., Garcia-Segura M.E., St-Pierre M.K., Savage J.C., Willis C., Picard K. (2024). Dark Microglia Are Abundant in Normal Postnatal Development, where they Remodel Synapses via Phagocytosis and Trogocytosis, and Are Dependent on TREM2. bioRxiv.

[157] St-Pierre M.K., Šimončičová E., Bögi E., Tremblay M.-È. (2020). Shedding light on the dark side of the microglia. ASN Neuro.

[158] Stephan A.H., Barres B.A., Stevens B. (2012). The complement system: An unexpected role in synaptic pruning during development and disease. Annu. Rev. Neurosci..

[159] Schafer D.P., Lehrman E.K., Kautzman A.G., Koyama R., Mardinly A.R., Yamasaki R., Ransohoff R.M., Greenberg M.E., Barres B.A., Stevens B. (2012). Microglia sculpt postnatal neural circuits in an activity and complement-dependent manner. Neuron.

[160] Duffy A.S., Eyo U.B. (2025). Microglia and Astrocytes in Postnatal Neural Circuit Formation. Glia.

[161] Scott-Hewitt N., Perrucci F., Morini R., Erreni M., Mahoney M., Witkowska A., Carey A., Faggiani E., Schuetz L.T., Mason S. (2020). Local externalization of phosphatidylserine mediates developmental synaptic pruning by microglia. EMBO J..

[162] Shen Y., Lue L.F., Yang L.B., Roher A., Kuo Y.M., Strohmeyer R., Goux W.J., Lee V., Johnson G.V., Webster S.D. (2001). Complement activation by neurofibrillary tangles in Alzheimer’s disease. Neurosci. Lett..

[163] Jiang H., Burdick D., Glabe C.G., Cotman C.W., Tenner A.J. (1994). beta-Amyloid activates complement by binding to a specific region of the collagen-like domain of the C1q A chain. J. Immunol..

[164] Rogers J., Cooper N.R., Webster S., Schultz J., McGeer P.L., Styren S.D., Civin W.H., Brachova L., Bradt B., Ward P. (1992). Complement activation by beta-amyloid in Alzheimer disease. Proc. Natl. Acad. Sci. USA.

[165] Zhong L., Sheng X., Wang W., Li Y., Zhuo R., Wang K., Zhang L., Hu D.-D., Hong Y., Chen L. (2023). TREM2 receptor protects against complement-mediated synaptic loss by binding to complement C1q during neurodegeneration. Immunity.

[166] Rueda-Carrasco J., Sokolova D., Lee S.E., Childs T., Jurčáková N., Crowley G., De Schepper S., Ge J.Z., Lachica J.I., Toomey C.E. (2023). Microglia-synapse engulfment via PtdSer-TREM2 ameliorates neuronal hyperactivity in Alzheimer’s disease models. EMBO J..

[167] Fracassi A., Marcatti M., Tumurbaatar B., Woltjer R., Moreno S., Taglialatela G. (2023). TREM2-induced activation of microglia contributes to synaptic integrity in cognitively intact aged individuals with Alzheimer’s neuropathology. Brain Pathol..

[168] Dejanovic B., Wu T., Tsai M.C., Graykowski D., Gandham V.D., Rose C.M., Bakalarski C.E., Ngu H., Wang Y., Pandey S. (2022). Complement C1q-dependent excitatory and inhibitory synapse elimination by astrocytes and microglia in Alzheimer’s disease mouse models. Nat. Aging.

[169] Das M., Mao W., Voskobiynyk Y., Necula D., Lew I., Petersen C., Zahn A., Yu G.Q., Yu X., Smith N. (2023). Alzheimer risk-increasing TREM2 variant causes aberrant cortical synapse density and promotes network hyperexcitability in mouse models. Neurobiol. Dis..

[170] Freria C.M., Brennan F.H., Sweet D.R., Guan Z., Hall J.C., Kigerl K.A., Nemeth D.P., Liu X., Lacroix S., Quan N. (2020). Serial Systemic Injections of Endotoxin (LPS) Elicit Neuroprotective Spinal Cord Microglia through IL-1-Dependent Cross Talk with Endothelial Cells. J. Neurosci..

[171] Cserép C., Pósfai B., Lénárt N., Fekete R., László Z.I., Lele Z., Orsolits B., Molnár G., Heindl S., Schwarcz A.D. (2020). Microglia monitor and protect neuronal function through specialized somatic purinergic junctions. Science.

[172] Inoue K., Tsuda M. (2018). Microglia in neuropathic pain: Cellular and molecular mechanisms and therapeutic potential. Nat. Rev. Neurosci..

[173] Tozaki-Saitoh H., Takeda H., Inoue K. (2022). The Role of Microglial Purinergic Receptors in Pain Signaling. Molecules.

[174] Tozaki-Saitoh H., Tsuda M., Miyata H., Ueda K., Kohsaka S., Inoue K. (2008). P2Y12 receptors in spinal microglia are required for neuropathic pain after peripheral nerve injury. J. Neurosci..

[175] Tsuda M., Masuda T., Kitano J., Shimoyama H., Tozaki-Saitoh H., Inoue K. (2009). IFN-γ receptor signaling mediates spinal microglia activation driving neuropathic pain. Proc. Natl. Acad. Sci. USA.

[176] Masuda T., Ozono Y., Mikuriya S., Kohro Y., Tozaki-Saitoh H., Iwatsuki K., Uneyama H., Ichikawa R., Salter M.W., Tsuda M. (2016). Dorsal horn neurons release extracellular ATP in a VNUT-dependent manner that underlies neuropathic pain. Nat. Commun..

[177] Boakye P.A., Tang S.J., Smith P.A. (2021). Mediators of Neuropathic Pain; Focus on Spinal Microglia, CSF-1, BDNF, CCL21, TNF-α, Wnt Ligands, and Interleukin 1β. Front. Pain Res..

[178] Sideris-Lampretsas G., Malcangio M. (2021). Microglial heterogeneity in chronic pain. Brain Behav. Immun..

[179] Yu X., Basbaum A., Guan Z. (2021). Contribution of colony-stimulating factor 1 to neuropathic pain. Pain Rep..

[180] Kohno K., Tsuda M. (2021). Role of microglia and P2X4 receptors in chronic pain. Pain Rep..

[181] Donnelly C.R., Andriessen A.S., Chen G., Wang K., Jiang C., Maixner W., Ji R.-R. (2020). Central nervous system targets: Glial cell mechanisms in chronic pain. Neurotherapeutics.

[182] Salter M.W., Beggs S. (2014). Sublime microglia: Expanding roles for the guardians of the CNS. Cell.

[183] Ferrini F., De Koninck Y. (2013). Microglia control neuronal network excitability via BDNF signalling. Neural Plast..

[184] Malcangio M., Sideris-Lampretsas G. (2025). How microglia contribute to the induction and maintenance of neuropathic pain. Nat. Rev. Neurosci..

[185] Long J., Tian G., He K., Su Y., Wang Z., Huang L., Yao Y., Li X., Lin Y. (2025). The role of microglia in neuropathic pain: A systematic review of animal experiments. Brain Res. Bull..

[186] Kobayashi M., Konishi H., Sayo A., Takai T., Kiyama H. (2016). TREM2/DAP12 signal elicits proinflammatory response in microglia and exacerbates neuropathic pain. J. Neurosci..

[187] Wang Y., Shi Y., Huang Y., Liu W., Cai G., Huang S., Zeng Y., Ren S., Zhan H., Wu W. (2020). Resveratrol mediates mechanical allodynia through modulating inflammatory response via the TREM2-autophagy axis in SNI rat model. J. Neuroinflamm..

[188] Yousefpour N., Locke S., Deamond H., Wang C., Marques L., St-Louis M., Ouellette J., Khoutorsky A., De Koninck Y., Ribeiro-da-Silva A. (2023). Time-dependent and selective microglia-mediated removal of spinal synapses in neuropathic pain. Cell Rep..

[189] Kohno K., Shirasaka R., Yoshihara K., Mikuriya S., Tanaka K., Takanami K., Inoue K., Sakamoto H., Ohkawa Y., Masuda T. (2022). A spinal microglia population involved in remitting and relapsing neuropathic pain. Science.

[190] Tsuda M., Masuda T., Kohno K. (2023). Microglial diversity in neuropathic pain. Trends Neurosci..

[191] Kamphuis W., Kooijman L., Schetters S., Orre M., Hol E.M. (2016). Transcriptional profiling of CD11c-positive microglia accumulating around amyloid plaques in a mouse model for Alzheimer’s disease. Biochim. Biophys. Acta (BBA) Mol. Basis Dis..

[192] Fiore N.T., Yin Z., Guneykaya D., Gauthier C.D., Hayes J.P., D’Hary A., Butovsky O., Moalem-Taylor G. (2021). Sex-specific transcriptome of spinal microglia in neuropathic pain due to peripheral nerve injury. Glia.

[193] Le Y., Tang S.Q., He W.Y., He J., Wang Y.H., Wang H. (2021). Contribution of Trem2 signaling to the development of painful diabetic neuropathy by mediating microglial polarization in mice. Res. Sq..

[194] Guruprasad B., Chaudhary P., Choedon T., Kumar V.L. (2015). Artesunate ameliorates functional limitations in Freund’s complete adjuvant-induced monoarthritis in rat by maintaining oxidative homeostasis and inhibiting COX-2 expression. Inflammation.

[195] Zhang L., Zhao Y., Gao T., Zhang H., Li J., Wang G., Wang C., Li Y. (2022). Artesunate reduces remifentanil-induced hyperalgesia and peroxiredoxin-3 hyperacetylation via modulating spinal metabotropic glutamate receptor 5 in rats. Neuroscience.

[196] Zhang L., Li N., Zhang H., Wang Y., Gao T., Zhao Y., Wang G., Yu Y., Wang C., Li Y. (2022). Artesunate therapy alleviates fracture-associated chronic pain after orthopedic surgery by suppressing CCL21-dependent TREM2/DAP12 inflammatory signaling in mice. Front. Pharmacol..

[197] Uzun T., Toptas O., Saylan A., Carver H., Turkoglu S.A. (2019). Evaluation and Comparison of the Effects of Artesunate, Dexamethasone, and Tacrolimus on Sciatic Nerve Regeneration. J. Oral Maxillofac. Surg..

[198] Ji X.T., Qian N.S., Zhang T., Li J.M., Li X.K., Wang P., Zhao D.S., Huang G., Zhang L., Fei Z. (2013). Spinal astrocytic activation contributes to mechanical allodynia in a rat chemotherapy-induced neuropathic pain model. PLoS ONE.

[199] Hu L.Y., Zhou Y., Cui W.Q., Hu X.M., Du L.X., Mi W.L., Chu Y.X., Wu G.C., Wang Y.Q., Mao-Ying Q.L. (2018). Triggering receptor expressed on myeloid cells 2 (TREM2) dependent microglial activation promotes cisplatin-induced peripheral neuropathy in mice. Brain Behav. Immun..

[200] Mannelli L.D.C., Pacini A., Micheli L., Tani A., Zanardelli M., Ghelardini C. (2014). Glial role in oxaliplatin-induced neuropathic pain. Exp. Neurol..

[201] Pevida M., Lastra A., Hidalgo A., Baamonde A., Menéndez L. (2013). Spinal CCL2 and microglial activation are involved in paclitaxel-evoked cold hyperalgesia. Brain Res. Bull..

[202] Zheng F., Xiao W.H., Bennett G. (2011). The response of spinal microglia to chemotherapy-evoked painful peripheral neuropathies is distinct from that evoked by traumatic nerve injuries. Neuroscience.

[203] Rotterman T.M., MacPherson K.P., Tansey M.G., Alvarez F.J. (2019). Motor circuit synaptic plasticity after peripheral nerve injury depends on a central neuroinflammatory response and a CCR2 mechanism. J. Neurosci..

[204] Rotterman T.M., Haley-Johnson Z., Pottorf T.S., Chopra T., Chang E., Zhang S., McCallum W.M., Fisher S., Franklin H., Alvarez M. (2024). Modulation of central synapse remodeling after remote peripheral injuries by the CCL2-CCR2 axis and microglia. Cell Rep..

[205] Rotterman T.M., García V.V., Housley S.N., Nardelli P., Sierra R., Fix C.E., Cope T.C. (2023). Structural preservation does not ensure function at sensory Ia–motoneuron synapses following peripheral nerve injury and repair. J. Neurosci..

[206] Rotterman T.M., Alvarez F.J. (2020). Microglia dynamics and interactions with motoneurons axotomized after nerve injuries revealed by two-photon imaging. Sci. Rep..

[207] Rotterman T.M., Akhter E.T., Lane A.R., MacPherson K.P., García V.V., Tansey M.G., Alvarez F.J. (2019). Spinal Motor Circuit Synaptic Plasticity after Peripheral Nerve Injury Depends on Microglia Activation and a CCR2 Mechanism. J. Neurosci..

[208] Manich G., Gómez-López A.R., Almolda B., Villacampa N., Recasens M., Shrivastava K., González B., Castellano B. (2020). Differential Roles of TREM2+ Microglia in Anterograde and Retrograde Axonal Injury Models. Front. Cell. Neurosci..

[209] Kobayashi M., Konishi H., Takai T., Kiyama H. (2015). A DAP12-dependent signal promotes pro-inflammatory polarization in microglia following nerve injury and exacerbates degeneration of injured neurons. Glia.

[210] Lieberman A. (1971). The axon reaction: A review of the principal features of perikaryal responses to axon injury. Int. Rev. Neurobiol..

[211] Fu S.Y., Gordon T. (1997). The cellular and molecular basis of peripheral nerve regeneration. Mol. Neurobiol..

[212] Castro R.W., Lopes M.C., De Biase L.M., Valdez G. (2024). Aging spinal cord microglia become phenotypically heterogeneous and preferentially target motor neurons and their synapses. Glia.

[213] García-Domínguez M. (2025). Pathological and Inflammatory Consequences of Aging. Biomolecules.

[214] Castro R.W., Lopes M.C., Settlage R.E., Valdez G. (2023). Aging alters mechanisms underlying voluntary movements in spinal motor neurons of mice, primates, and humans. JCI Insight.

[215] You J., Youssef M.M., Santos J.R., Lee J., Park J. (2023). Microglia and astrocytes in amyotrophic lateral sclerosis: Disease-associated states, pathological roles, and therapeutic potential. Biology.

[216] Clarke B.E., Patani R. (2020). The microglial component of amyotrophic lateral sclerosis. Brain J. Neurol..

[217] Brites D., Vaz A.R. (2014). Microglia centered pathogenesis in ALS: Insights in cell interconnectivity. Front. Cell. Neurosci..

[218] Henkel J.S., Beers D.R., Zhao W., Appel S.H. (2009). Microglia in ALS: The good, the bad, and the resting. J. Neuroimmune Pharmacol..

[219] Dols-Icardo O., Montal V., Sirisi S., López-Pernas G., Cervera-Carles L., Querol-Vilaseca M., Muñoz L., Belbin O., Alcolea D., Molina-Porcel L. (2020). Motor cortex transcriptome reveals microglial key events in amyotrophic lateral sclerosis. Neurol. Neuroimmunol. Neuroinflamm..

[220] Chen L.X., Zhang M.D., Xu H.F., Ye H.Q., Chen D.F., Wang P.S., Bao Z.W., Zou S.M., Lv Y.T., Wu Z.Y. (2024). Single-Nucleus RNA Sequencing Reveals the Spatiotemporal Dynamics of Disease-Associated Microglia in Amyotrophic Lateral Sclerosis. Research.

[221] Tam O.H., Rozhkov N.V., Shaw R., Kim D., Hubbard I., Fennessey S., Propp N., Phatnani H., Kwan J., Sareen D. (2019). Postmortem Cortex Samples Identify Distinct Molecular Subtypes of ALS: Retrotransposon Activation, Oxidative Stress, and Activated Glia. Cell Rep..

[222] Jauregui C., Blanco-Luquin I., Macías M., Roldan M., Caballero C., Pagola I., Mendioroz M., Jericó I. (2023). Exploring the Disease-Associated microglia state in amyotrophic lateral sclerosis. Biomedicines.

[223] Maniatis S., Äijö T., Vickovic S., Braine C., Kang K., Mollbrink A., Fagegaltier D., Andrusivová Ž., Saarenpää S., Saiz-Castro G. (2019). Spatiotemporal dynamics of molecular pathology in amyotrophic lateral sclerosis. Science.

[224] Sreedharan J., Blair I.P., Tripathi V.B., Hu X., Vance C., Rogelj B., Ackerley S., Durnall J.C., Williams K.L., Buratti E. (2008). TDP-43 Mutations in Familial and Sporadic Amyotrophic Lateral Sclerosis. Science.

[225] Prasad A., Bharathi V., Sivalingam V., Girdhar A., Patel B.K. (2019). Molecular mechanisms of TDP-43 misfolding and pathology in amyotrophic lateral sclerosis. Front. Mol. Neurosci..

[226] Scotter E.L., Chen H.J., Shaw C.E. (2015). TDP-43 proteinopathy and ALS: Insights into disease mechanisms and therapeutic targets. Neurotherapeutics.

[227] Waters R., Adkins R., Yakura J. (1991). Definition of complete spinal cord injury. Spinal Cord..

[228] Stauffer E.S. (1975). Diagnosis and prognosis of acute cervical spinal cord injury. Clin. Orthop. Relat. Res..

[229] Klose K.J., Green B.A., Smith R.S., Adkins R.H., MacDonald A.M. (1980). University of Miami Neuro-Spinal Index (UMNI): A quantitative method for determining spinal cord function. Paraplegia.

[230] Maynard F.M., Bracken M.B., Creasey G., Ditunno J.F., Donovan W.H., Ducker T.B., Garber S.L., Marino R.J., Stover S.L., Tator C.H. (1997). International standards for neurological and functional classification of spinal cord injury. Spinal Cord.

[231] Cheriyan T., Ryan D., Weinreb J., Cheriyan J., Paul J., Lafage V., Kirsch T., Errico T. (2014). Spinal cord injury models: A review. Spinal Cord.

[232] Hall M. (1840). Second Memoir on some principles of the pathology of the nervous system. Med. Chir. Trans..

[233] Ko H.Y. (2018). Revisit Spinal Shock: Pattern of Reflex Evolution during Spinal Shock. Korean J. Neurotrauma.

[234] Bastian H.C. (1890). On the symptomatology of total transverse lesions of the spinal cord; with special reference to the condition of the various reflexes. Med. Chir. Trans..

[235] Silva N.A., Sousa N., Reis R.L., Salgado A.J. (2014). From basics to clinical: A comprehensive review on spinal cord injury. Prog. Neurobiol..

[236] Lindan R., Joiner E., Freehafer A., Hazel C. (1980). Incidence and clinical features of autonomic dysreflexia in patients with spinal cord injury. Spinal Cord.

[237] Widerström-Noga E.G., Felipe-Cuervo E., Yezierski R.P. (2001). Relationships among clinical characteristics of chronic pain after spinal cord injury. Arch. Phys. Med. Rehabil..

[238] Oyinbo C. (2011). Secondary injury mechanisms in traumatic spinal cord injury: A nugget of this multiply cascade. Acta Neurobiol. Exp..

[239] Ahuja C.S., Wilson J.R., Nori S., Kotter M., Druschel C., Curt A., Fehlings M.G. (2017). Traumatic spinal cord injury. Nat. Rev. Dis. Primers.

[240] Tator C.H., Fehlings M.G. (1991). Review of the secondary injury theory of acute spinal cord trauma with emphasis on vascular mechanisms. J. Neurosurg..

[241] Brennan F.H., Li Y., Wang C., Ma A., Guo Q., Li Y., Pukos N., Campbell W.A., Witcher K.G., Guan Z. (2022). Microglia coordinate cellular interactions during spinal cord repair in mice. Nat. Commun..

[242] Swarts E.A., Brennan F.H. (2025). Evolving insights on the role of microglia in neuroinflammation, plasticity, and regeneration of the injured spinal cord. Front. Immunol..

[243] Kroner A., Almanza J.R. (2019). Role of microglia in spinal cord injury. Neurosci. Lett..

[244] Zhou X., He X., Ren Y. (2014). Function of microglia and macrophages in secondary damage after spinal cord injury. Neural Regen. Res..

[245] Brockie S., Hong J., Fehlings M.G. (2021). The role of microglia in modulating neuroinflammation after spinal cord injury. Int. J. Mol. Sci..

[246] David S., Kroner A. (2011). Repertoire of microglial and macrophage responses after spinal cord injury. Nat. Rev. Neurosci..

[247] Xu L., Wang J., Ding Y., Wang L., Zhu Y.J. (2022). Current knowledge of microglia in traumatic spinal cord injury. Front. Neurol..

[248] Brockie S., Zhou C., Fehlings M.G. (2024). Resident immune responses to spinal cord injury: Role of astrocytes and microglia. Neural Regen. Res..

[249] Zha X., Zheng G., Skutella T., Kiening K., Unterberg A., Younsi A. (2025). Microglia: A promising therapeutic target in spinal cord injury. Neural Regen. Res..

[250] Zhao T., Di J., Kang Y., Zhang H., Yao S., Liu B., Rong L. (2025). TREM2 Impedes Recovery After Spinal Cord Injury by Regulating Microglial Lysosomal Membrane Permeabilization-Mediated Autophagy.

[251] Burgess M., Wicks K., Gardasevic M., Mace K.A. (2019). Cx3CR1 expression identifies distinct macrophage populations that contribute differentially to inflammation and repair. Immunohorizons.

[252] Lee M., Lee Y., Song J., Lee J., Chang S.Y. (2018). Tissue-specific role of CX3CR1 expressing immune cells and their relationships with human disease. Immune Netw..

[253] Jung S., Aliberti J., Graemmel P., Sunshine M.J., Kreutzberg G.W., Sher A., Littman D.R. (2000). Analysis of fractalkine receptor CX3CR1 function by targeted deletion and green fluorescent protein reporter gene insertion. Mol. Cell. Biol..

[254] Flores A.J., Lavemia C., Owens P.W. (2000). Anatomy and physiology of peripheral nerve injury and repair. Am. J. Orthop. Belle Mead.

[255] Geuna S., Raimondo S., Ronchi G., Di Scipio F., Tos P., Czaja K., Fornaro M. (2009). Histology of the peripheral nerve and changes occurring during nerve regeneration. Int. Rev. Neurobiol..

[256] Boissaud-Cooke M., Pidgeon T.E., Tunstall R., Tubbs R.S., Rizk E., Shoja M.M., Loukas M., Barbaro N., Spinner R.J. (2015). Chapter 37—The Microcirculation of Peripheral Nerves: The Vasa Nervorum. Nerves and Nerve Injuries.

[257] Appenzeller O., Dhital K.K., Cowen T., Burnstock G. (1984). The nerves to blood vessels supplying blood to nerves: The innervation of vasa nervorum. Brain Res..

[258] Sladjana U.Z., Ivan J.D., Bratislav S.D. (2008). Microanatomical structure of the human sciatic nerve. Surg. Radiol. Anat..

[259] Brosius Lutz A., Lucas T.A., Carson G.A., Caneda C., Zhou L., Barres B.A., Buckwalter M.S., Sloan S.A. (2022). An RNA-sequencing transcriptome of the rodent Schwann cell response to peripheral nerve injury. J. Neuroinflamm..

[260] Gerber D., Pereira J.A., Gerber J., Tan G., Dimitrieva S., Yángüez E., Suter U. (2021). Transcriptional profiling of mouse peripheral nerves to the single-cell level to build a sciatic nerve ATlas (SNAT). eLife.

[261] Wang P.L., Yim A.K., Kim K.W., Avey D., Czepielewski R.S., Colonna M., Milbrandt J., Randolph G.J. (2020). Peripheral nerve resident macrophages share tissue-specific programming and features of activated microglia. Nat. Commun..

[262] Wang P.L. (2021). Peripheral Nerve Macrophages and Their Implications in Neuroimmunity.

[263] Tsuchiya T., Miyawaki S., Teranishi Y., Ohara K., Hirano Y., Ogawa S., Torazawa S., Sakai Y., Hongo H., Ono H. (2025). Current molecular understanding of central nervous system schwannomas. Acta Neuropathol. Commun..

[264] Baruah P., Mahony C., Marshall J.L., Smith C.G., Monksfield P., Irving R.I., Dumitriu I.E., Buckley C.D., Croft A.P. (2024). Single-cell RNA sequencing analysis of vestibular schwannoma reveals functionally distinct macrophage subsets. Br. J. Cancer.

[265] Katzenelenbogen Y., Sheban F., Yalin A., Yofe I., Svetlichnyy D., Jaitin D.A., Bornstein C., Moshe A., Keren-Shaul H., Cohen M. (2020). Coupled scRNA-Seq and intracellular protein activity reveal an immunosuppressive role of TREM2 in cancer. Cell.

[266] Nakamura K., Smyth M.J. (2020). TREM2 marks tumor-associated macrophages. Signal Transduct. Target. Ther..

[267] Fu Y., Zhu Y., Guo L., Liu Y. (2023). Identification of key genes and immune infiltration based on weighted gene co-expression network analysis in vestibular schwannoma. Medicine.

[268] Olsson Y., Sjöstrand J. (1969). Origin of macrophages in Wallerian degeneration of peripheral nerves demonstrated autoradiographically. Exp. Neurol..

[269] Monaco S., Gehrmann J., Raivich G., Kreutzberg G.W. (1992). MHC-positive, ramified macrophages in the normal and injured rat peripheral nervous system. J. Neurocytol..

[270] Chen P., Piao X., Bonaldo P. (2015). Role of macrophages in Wallerian degeneration and axonal regeneration after peripheral nerve injury. Acta Neuropathol..

[271] Cattin A.L., Burden J.J., Van Emmenis L., Mackenzie F.E., Hoving J.J., Calavia N.G., Guo Y., McLaughlin M., Rosenberg L.H., Quereda V. (2015). Macrophage-induced blood vessels guide Schwann cell-mediated regeneration of peripheral nerves. Cell.

[272] Gordon T. (2020). Peripheral Nerve Regeneration and Muscle Reinnervation. Int. J. Mol. Sci..

[273] Chen B., Chen Q., Parkinson D.B., Dun X.P. (2019). Analysis of Schwann cell migration and axon regeneration following nerve injury in the sciatic nerve bridge. Front. Mol. Neurosci..

[274] Witzel C., Rohde C., Brushart T.M. (2005). Pathway sampling by regenerating peripheral axons. J. Comp. Neurol..

[275] Tomlinson J.E., Žygelytė E., Grenier J.K., Edwards M.G., Cheetham J. (2018). Temporal changes in macrophage phenotype after peripheral nerve injury. J. Neuroinflamm..

[276] Rios R., Jablonka-Shariff A., Broberg C., Snyder-Warwick A.K. (2021). Macrophage roles in peripheral nervous system injury and pathology: Allies in neuromuscular junction recovery. Mol. Cell. Neurosci..

[277] Ydens E., Cauwels A., Asselbergh B., Goethals S., Peeraer L., Lornet G., Almeida-Souza L., Van Ginderachter J.A., Timmerman V., Janssens S. (2012). Acute injury in the peripheral nervous system triggers an alternative macrophage response. J. Neuroinflamm..

[278] Shiraishi W., Yamasaki R., Hashimoto Y., Ko S., Kobayakawa Y., Isobe N., Matsushita T., Kira J.I. (2021). Clearance of peripheral nerve misfolded mutant protein by infiltrated macrophages correlates with motor neuron disease progression. Sci. Rep..

[279] Graber D.J., Hickey W.F., Harris B.T. (2010). Progressive changes in microglia and macrophages in spinal cord and peripheral nerve in the transgenic rat model of amyotrophic lateral sclerosis. J. Neuroinflamm..

[280] Kano O., Beers D.R., Henkel J.S., Appel S.H. (2012). Peripheral nerve inflammation in ALS mice: Cause or consequence. Neurology.

[281] Martínez-Muriana A., Mancuso R., Francos-Quijorna I., Olmos-Alonso A., Osta R., Perry V.H., Navarro X., Gomez-Nicola D., López-Vales R. (2016). CSF1R blockade slows the progression of amyotrophic lateral sclerosis by reducing microgliosis and invasion of macrophages into peripheral nerves. Sci. Rep..

[282] Chiot A., Zaïdi S., Iltis C., Ribon M., Berriat F., Schiaffino L., Jolly A., de la Grange P., Mallat M., Bohl D. (2020). Modifying macrophages at the periphery has the capacity to change microglial reactivity and to extend ALS survival. Nat. Neurosci..

[283] Msheik Z., El Massry M., Rovini A., Billet F., Desmoulière A. (2022). The macrophage: A key player in the pathophysiology of peripheral neuropathies. J. Neuroinflamm..

[284] MacIntosh B.R., Gardiner P.F., McComas A.J. (2006). Skeletal Muscle: Form and Function.

[285] Lieber R.L. (2002). Skeletal Muscle Structure, Function, and Plasticity.

[286] Liddel E., Sherrington C. (1924). Reflexes in response to stretch (myotatic reflexes). Proc. R. Soc. Lond. Ser. B.

[287] Eccles J., Eccles R.M., Lundberg A. (1957). Synaptic actions on motoneurones caused by impulses in Golgi tendon organ afferents. J. Physiol..

[288] Mendell L.M., Henneman E. (1971). Terminals of single Ia fibers: Location, density, and distribution within a pool of 300 homonymous motoneurons. J. Neurophysiol..

[289] Bączyk M., Manuel M., Roselli F., Zytnicki D. (2022). Diversity of Mammalian Motoneurons and Motor Units. Vertebrate Motoneurons.

[290] Kernell D. (2006). The Motoneurone and its Muscle Fibres.

[291] Manuel M., Zytnicki D. (2011). Alpha, beta and gamma motoneurons: Functional diversity in the motor system’s final pathway. J. Integr. Neurosci..

[292] Alhindi A., Boehm I., Forsythe R.O., Miller J., Skipworth R.J.E., Simpson H., Jones R.A., Gillingwater T.H. (2021). Terminal Schwann cells at the human neuromuscular junction. Brain Commun..

[293] Balice-Gordon R.J. (1996). Schwann cells: Dynamic roles at the neuromuscular junction. Curr. Biol..

[294] Hastings R.L., Valdez G. (2024). Origin, identity, and function of terminal Schwann cells. Trends Neurosci..

[295] Hastings R.L., Mikesh M., Lee Y.i., Thompson W.J. (2020). Morphological remodeling during recovery of the neuromuscular junction from terminal Schwann cell ablation in adult mice. Sci. Rep..

[296] Muir A., Kanji A., Allbrook D. (1965). The structure of the satellite cells in skeletal muscle. J. Anat..

[297] Mauro A. (1961). Satellite cell of skeletal muscle fibers. J. Biophys. Biochem. Cytol..

[298] Rubenstein A.B., Smith G.R., Raue U., Begue G., Minchev K., Ruf-Zamojski F., Nair V.D., Wang X., Zhou L., Zaslavsky E. (2020). Single-cell transcriptional profiles in human skeletal muscle. Sci. Rep..

[299] De Micheli A.J., Spector J.A., Elemento O., Cosgrove B.D. (2020). A reference single-cell transcriptomic atlas of human skeletal muscle tissue reveals bifurcated muscle stem cell populations. Skelet. Muscle.

[300] Kang H., Tian L., Mikesh M., Lichtman J.W., Thompson W.J. (2014). Terminal Schwann Cells Participate in Neuromuscular Synapse Remodeling during Reinnervation following Nerve Injury. J. Neurosci..

[301] Reynolds M., Woolf C. (1992). Terminal Schwann cells elaborate extensive processes following denervation of the motor endplate. J. Neurocytol..

[302] Bermedo-García F., Zelada D., Martínez E., Tabares L., Henríquez J.P. (2022). Functional regeneration of the murine neuromuscular synapse relies on long-lasting morphological adaptations. BMC Biol..

[303] Son Y.J., Thompson W.J. (1995). Schwann cell processes guide regeneration of peripheral axons. Neuron.

[304] Yin P., Chen M., Rao M., Lin Y., Zhang M., Xu R., Hu X., Chen R., Chai W., Huang X. (2024). Deciphering immune landscape remodeling unravels the underlying mechanism for synchronized muscle and bone aging. Adv. Sci..

[305] Larouche J.A., Wallace E.C., Spence B.D., Buras E., Aguilar C.A. (2023). Spatiotemporal mapping of immune and stem cell dysregulation after volumetric muscle loss. JCI Insight.

[306] Wang Y., Wang X., Alabdullatif S., Homma S.T., Alekseyev Y.O., Zhou L. (2025). Expansion and pathogenic activation of skeletal muscle–resident macrophages in mdx5cv/Ccr2−/− mice. Proc. Natl. Acad. Sci. USA.

[307] Essex A.L., Huot J.R., Deosthale P., Wagner A., Figueras J., Davis A., Damrath J., Pin F., Wallace J., Bonetto A. (2022). Triggering Receptor Expressed on Myeloid Cells 2 (TREM2) R47H Variant Causes Distinct Age- and Sex-Dependent Musculoskeletal Alterations in Mice. J. Bone Min. Res..

[308] Tacconi S., Giudetti A.M., Blangero F., Meugnier E., El-jaafari A., Longo S., Angilé F., Fanizzi F.P., Canaple L., Jalabert A. (2025). LAM/TREM2^+^ macrophages release extracellular vesicles and extracellular lipid droplets which modulate the phenotype of recipient macrophages and homeostasis of skeletal muscle cells. bioRxiv.

[309] Lu C.-Y., Santosa K.B., Jablonka-Shariff A., Vannucci B., Fuchs A., Turnbull I., Pan D., Wood M.D., Snyder-Warwick A.K. (2020). Macrophage-derived vascular endothelial growth factor-A is integral to neuromuscular junction reinnervation after nerve injury. J. Neurosci..

[310] Van Dyke J.M., Smit-Oistad I.M., Macrander C., Krakora D., Meyer M.G., Suzuki M. (2016). Macrophage-mediated inflammation and glial response in the skeletal muscle of a rat model of familial amyotrophic lateral sclerosis (ALS). Exp. Neurol..

[311] Chiu I.M., Phatnani H., Kuligowski M., Tapia J.C., Carrasco M.A., Zhang M., Maniatis T., Carroll M.C. (2009). Activation of innate and humoral immunity in the peripheral nervous system of ALS transgenic mice. Proc. Natl. Acad. Sci. USA.

[312] Dachs E., Hereu M., Piedrafita L., Casanovas A., Calderó J., Esquerda J.E. (2011). Defective neuromuscular junction organization and postnatal myogenesis in mice with severe spinal muscular atrophy. J. Neuropathol. Exp. Neurol..

[313] Deczkowska A., Weiner A., Amit I. (2020). The Physiology, Pathology, and Potential Therapeutic Applications of the TREM2 Signaling Pathway. Cell.

[314] Chtarto A., Humbert-Claude M., Bockstael O., Das A.T., Boutry S., Breger L.S., Klaver B., Melas C., Barroso-Chinea P., Gonzalez-Hernandez T. (2016). A regulatable AAV vector mediating GDNF biological effects at clinically-approved sub-antimicrobial doxycycline doses. Mol. Ther. Methods Clin. Dev..

[315] Zhou L., Wang Y., Xu Y., Zhang Y., Zhu C. (2024). A comprehensive review of AAV-mediated strategies targeting microglia for therapeutic intervention of neurodegenerative diseases. J. Neuroinflamm..

[316] Masoudi N., Willen J., Daniels C., Jenkins B.A., Furber E.C., Kothiya M., Banjoko M.B., Gowda R., Hendricks J., Fang Y.-Y. (2024). Microglial-targeted Gene Therapy: Developing a Disease Modifying Treatment for ALSP Associated with CSF1R Mutations (ALSP-CSF1R) (P11-4.012). Neurology.

